# Enhancing wellbeing in medical practice: Exploring interventions and effectiveness for improving the work lives of resident (junior) doctors: A systematic review and narrative synthesis

**DOI:** 10.1016/j.fhj.2024.100195

**Published:** 2024-10-16

**Authors:** Yuri Hirayama, Sunera Khan, Charn Gill, Maxwell Thoburn, Jennifer Hancox, Jameel Muzaffar

**Affiliations:** University Hospitals Birmingham NHS Foundation Trust, Queen Elizabeth Hospital Birmingham, Edgbaston, Birmingham, B15 2TT

**Keywords:** Junior doctors, Doctors in training, Resident doctors, Residents, Wellbeing interventions, Work-life balance, Mental health, Healthcare Workforce Retention, Post-COVID Healthcare

## Abstract

**Introduction:**

Globally, resident doctors face challenges like long work hours, critical decision-making stress, and exposure to death and distress, prompting concern for their wellbeing. This study addresses the need for interventions to improve their working conditions, vital for enhancing quality of life, patient care and retaining a skilled workforce.

**Methods:**

Following PRISMA guidelines, a systematic literature review until 3 January 2024 explored interventions for resident Ddoctors pre- and post-COVID-19. It evaluated intervention effectiveness, metrics and feasibility, excluding studies with high bias risk.

**Results:**

The review identified diverse interventions, from mentoring to wellness resources, showing significant improvements in job satisfaction, mental health and professional growth among resident doctors. Due to methodological variations, a narrative synthesis was conducted.

**Conclusion:**

Effective interventions addressing resident doctors’ challenges can notably enhance their wellbeing and job satisfaction. Scaling such interventions is vital for fostering supportive work environments, sustaining the healthcare workforce and improving patient care quality.

## Highlights

• Diverse interventions improve Resident Doctors' well-being and job satisfaction.
• Mentorship and social support significantly reduce burnout and enhance professional growth.
• Mindfulness and physical exercise interventions show mixed but valuable subjective benefits.
• Enhanced workplace support addresses key stressors and improves mental health.
• Reducing work hours alone is insufficient; comprehensive support systems are crucial.

## Introduction

A career in medicine can be rewarding and intellectually stimulating, balanced against long work hours, regularly making tough decisions amid uncertainty, and the responsibility of navigating death and distress while maintaining compassion.^1^^,^^2^ The training phase is notoriously challenging for resident doctors,^3^ encompassing individuals from their first job beyond medical school until they reach a position as a consultant or GP.

Resident doctors grapple with competitive job applications, the financial burden of numerous mandatory examinations (each costing more than £500 on average), and adapting to frequent changes in job, role, team and hospital settings. This is against a background of poor work–life balance, poor pay, lack of autonomy, inadequate senior support and prolonged working hours.^4^, ^5^, ^6^ The pandemic further exacerbated these challenges, bringing to light the critical need for effective support systems and interventions to safeguard the mental health and job satisfaction of healthcare workers, particularly those in the early stages of their carers.^7^^,^^8^ Unsurprisingly, there is increasing concern about diminished morale among resident doctors.^9^^,^^10^ Increased levels of burnout, stress and psychiatric morbidity^11^ are identified as significant contributors to a notably high number of suicides.^6^^,^^12^, ^13^, ^14^, ^15^

Given that previous systematic reviews have focused on literature published before the COVID-19 era, with the UK review limited to doctors within 5 years of graduation^12^ and others concentrating solely on burnout,^16^ a new systematic review is required to evaluate more recent data. This will provide a comprehensive understanding of the current effectiveness of wellbeing interventions for resident doctors, considering the unique challenges posed by the pandemic and the post-pandemic era and addressing a broader range of outcomes beyond burnout.

## Methods

### Protocol registration and search strategy

A systematic literature review was conducted in accordance with the Preferred Reporting Items for Systematic Reviews and Meta-Analyses (PRISMA) guidelines.^17^ The protocol was prospectively registered on Open Science Framework (DOI 10.17605/OSF.IO/FC6JD). There have been no modifications made since the registration.

In consultation with a librarian trained in MeSH terms and literature search, a comprehensive search was conducted in Embase, Medline, CINAHL, HMIC, PubMed and the Cochrane Database on 3January 2024. The full search strategy is detailed in Appendix 1. The inclusion criteria encompassed all primary research articles published in the English language before 3 January 2024. This included only randomised controlled trials (RCTs), case controls, cohort studies, and case studies from all geographical areas. The primary focus of these papers was on interventions and their effectiveness in improving the work lives of resident doctors. Exclusions comprised conference abstracts, letters to editors, commentaries, and articles in languages other than English. The main search terms used (non-consultant, junior doctor, doctor-in-training) are not classified as MeSH headings in the databases. Combining these terms with others (eg intervention*) runs the risk of returning few papers containing the desired free-text terms. To minimise the risk of missing relevant papers in the initial search, the references of the first set of identified papers were screened to find additional relevant studies and compile a list of included papers. The references of these included papers were then reviewed once more to identify further relevant studies.

### Assessment of risk of bias

Risk of bias assessment was performed by one reviewer (YH) using the Cochrane Risk of Bias 2 (RoB 2) tool^18^ for randomised control trials (Supplementary Table 2), Risk Of Bias In Non-randomised Studies of Exposure (ROBINS-E)^19^ for observational studies of exposures (Supplementary Table 3), Appraisal tool for Cross-Sectional Studies (AXIS)^20^ for cross sectional studies (Supplementary Table 4), and Critical Skills Appraisal Programme (CASP) Qualitative Studies Checklist^21^ for qualitative studies (Supplementary Table 5). The authors deliberated on bias assessment, and any studies identified as having a high risk of bias were subsequently excluded.

### Data extraction and analysis

The following datapoints were collected directly from each paper by one reviewer (YH):

1. Author names
2. Publication year
3. Publication journal
4. Conflicts of interest
5. Sources of funding
6. Study sample characteristics: population being studied, participant inclusion/exclusion criteria, number and age of participants
7. Number of withdrawals/dropouts
8. Intervention: type and duration
9. Main outcomes
10. Study limitations
11. Level of evidence, scored using the Oxford Centre for Evidence-Based Medicine (CEBM) criteria, where level 1 is the highest level of evidence^22^

Due methodological heterogeneity meta-analysis was not undertaken. Instead, a narrative synthesis is presented.

## Results

**Fig. 1:**
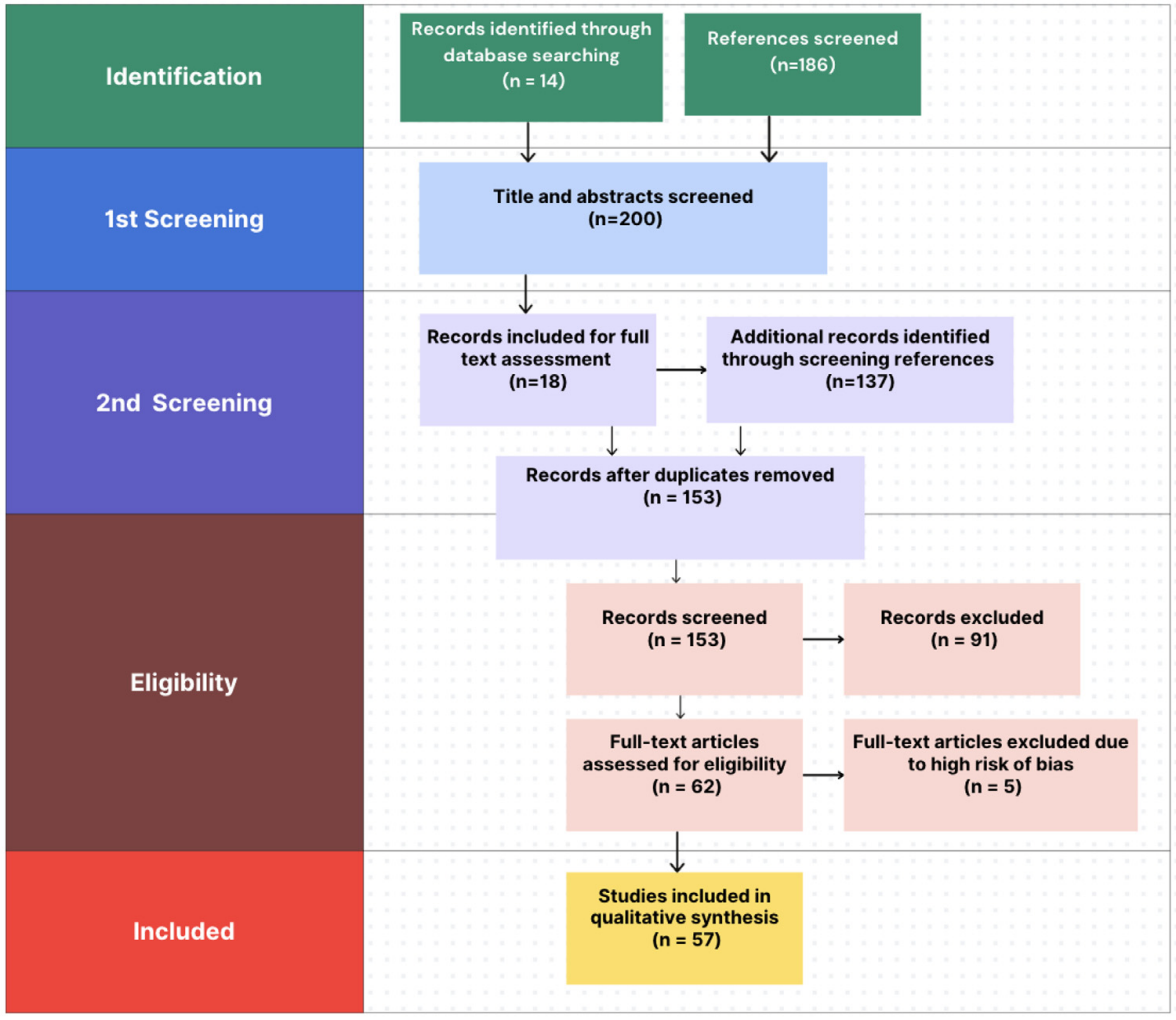
PRISMA flow diagram.

Electronic searches identified 14 papers. Screening the reference lists (n=186) using the snowball methodology resulted in a total of 200 papers (Fig. 1). After screening titles and abstracts, 18 papers remained, with duplicates, conference abstracts and studies lacking full texts excluded. Screening the reference lists of these papers again identified 137 additional potentially relevant papers. After excluding duplicates, 153 papers were available for screening. Sixty-two texts underwent full-text screening. Ultimately, 57 papers were included in the final analysis (Table 1, Supplementary Table 1). Sample sizes ranged from seven to 628. Most study designs were RCTs (22/57, 38 %) and pre-post studies (17/57, 29 %). 32 studies (56 %) were performed in the USA, eight studies in the UK (14 %) and five in Australia (9 %). The highest number of papers focused on trainees in internal medicine (17/57, 30 %), surgery (9/57, 16 %) and paediatrics (9/57, 16 %). The most common age group was late 20s to 30s. Findings are presented in the following seven categories.

**Table 1:** Literature on interventions and their effectiveness in enhancing the work lives of non-consultant doctors, summarised into thematic categories of intervention.

Author	Country	Study type	Population specialty	Sample size	Intervention details	Outcome measure (positive effectiveness)	CEBM level of evidence^22^
Mentorship and social support							

Rogers *et al*^24^	Canada	Cross-sectional	Medicine	198	Support from family, friends and colleagues	Burnout and loneliness (yes)	4
Webb *et al*^28^	UK	Pre-post study	Medicine	23	Peer mentorship for 1 year	Attitude to work (yes), stress management (yes), acquisition of transferable skills (yes)	3
Eisen *et al*^25^	UK	Pre-post study	Paediatrics	18	Peer mentorship for 1 year	Attitude to work (yes), work–life balance (yes), confidence (yes)	3
Hsu *et al*^23^	USA	Cross-sectional	ENT	47	Faculty mentorship	Satisfaction with training (yes)	4
Prins *et al*^27^	Netherlands	Cross-sectional	Medicine	158	Support from supervisors, fellow medical residents, nurses and patients	Burnout (yes), depersonalisation (yes)	4
Feld *et al*^26^	UK	Cross-sectional	Medicine	58	Support group	Sense of solidarity (yes)	4
Workshops and group discussions on resilience, stress management and skills to sustain wellbeing							

Rich *et al*^29^	UK	Pre-post study	Medicine	22	Six 2-hour workshops, led by a health psychologist and a human–computer interaction specialist, provide guidance on self-care techniques, self-compassion, and digital wellbeing strategies.	Found helpful (yes), burnout (yes), boundary control (yes), wellbeing (no)	3
Axisa *et al*^30^	Australia	RCT	Medicine, paediatrics	59	4.5-hour workshop on understanding wellbeing and resilience, mindfulness, barriers to looking after wellbeing, giving, and receiving feedback and stress management strategies	Found helpful (yes), reduction in alcohol use and depression (no), burnout (no)	3
Mache *et al*^37^	Germany	RCT	Medicine	80	Twelve weekly 1.5 h of psychosocial competency training on work related self-care strategies, problem solving skills and solution-focused counselling.	Reduction in work-related stress (yes), emotional exhaustion (yes), improved emotional regulation (yes), job satisfaction (no)	2
Mache *et al*^36^	Germany	RCT	Emergency medicine	70	90-minute sessions over 3 months on problem-focused and emotion-focused coping skills, cognitive behavioural, and solution-focused counselling in team training	Reduction in work-related stress (yes), emotional exhaustion (yes), perceived stress (yes)	2
Ripp *et al*^33^	USA	RCT	Medicine	51	Twice-monthly workshops with trained discussion group leaders to discuss topics related to stress, balance, burnout and job satisfaction.	Burnout (no), depersonalisation (no)	2
Maher *et al*^35^	USA	Cohort study	Surgery	26	Three 3-hour workshops on stressor identification and stress management techniques.	Found helpful (yes), improvement in objective performance (no), anxiety levels (no)	3
Saadat *et al*^32^	USA	RCT	Anaesthetics	58	16 weekly workshop on eliminating or modifying sources of stress, use of effective problem-solving and communication skills, minimising the use of avoidance coping	Social support (yes), problem solving coping (yes), less anxiety (yes), alcohol consumption (no), avoidance coping (no)	2
Martins *et al*^31^	Argentina	RCT	Paediatrics	74	2.5-hour workshop addressing the repercussions of burnout syndrome on professional activity, recognition of risk indicators for burnout syndrome, and providing coping tools	Depersonalisation (yes), personal accomplishment (no), emotional exhaustion (no)	3
Winkel *et al*^34^	USA	Cohort study	Obstetrics and gynaecology	18	Residents generated a list of topics they felt were important to them that fell outside of their standard curriculum. Six 1-hour reflective writing workshops on each topic.	Found helpful (yes), burnout (no), empathy (no)	3
Bragard *et al*^38^	Belgium	RCT	Medicine	96	A 40-hour training programme emphasising communication skills in cancer care, consisting of a 30-hour segment dedicated to communication skills and a separate 10-hour module focusing on stress management.	Self-efficacy (yes), reduction in stress to communication (yes), burnout (no)	3
McCue *et al*^39^	USA	Cohort study	Medicine, paediatrics	64	4-hour workshop on effective strategies for managing stress, personal management skills, relationship skills, outlook skills and stamina skills.	Decreased emotional exhaustion (yes), ability to cope with pressure (yes), self-esteem (yes)	3
Mindfulness							

Fraiman *et al*^47^	USA	RCT	Paediatrics	340	Monthly 7-hour sessions of mindfulness interspersed with refresher classes.	Burnout (no), emotional exhaustion (no), empathy (no)	2
Fendel *et al*^44^	Germany	RCT	Medicine	147	Eight weekly 135 min session on mindfulness delivered by psychiatrists.	Burnout (yes), less perceived job strain (yes), attentiveness (yes), depression (no), anxiety (no), error (no), cortisol level (no)	2
Lebares *et al*^48^	USA	RCT	Mixed	89	8 weekly 2-hour mindfulness classes explicitly applied to hospital-based work, and challenges of maintaining wellbeing during demanding training.	Stress (no), emotional exhaustion (yes), depersonalisation (yes), performance (yes)	2
Cheung *et al*^42^	USA	RCT	Medicine	26	12 min video of simulation-based mastery learning (SBML) programme for medical residents with a brief mindfulness intervention aimed to reduce procedural stress and improve simulator performance	Procedural stress and performance (yes)	3
Minichiello *et al*^51^	USA	Pre-post study	Family medicine (GP)	17	10 hour mindfulness training over 2 months, co-taught by MBSR teachers and family physicians	Found helpful (yes), stress (yes)	3
Forbes *et al*^53^	Australia	Cohort study	Intern (foundation)	53	Four 90-min group mindfulness and meditation sessions delivered by psychiatrists.	Found helpful (yes)	4
Bu *et al*^40^	UK	Pre-post study	Intern (foundation)	20	Six weekly 2 h mindfulness sessions delivered by Breathwork	Reduction in anxiety (yes), stress (yes), improved sleep quality (yes), relationship with patients (yes)	3
Lebares *et al*^45^	USA	RCT	Surgery	19	Eight weekly 2 hour modified mindfulness-based stress reduction classes	Stress (yes), memory (yes), surgical skills (yes)	3
Bentley *et al*^57^	USA	Pre-post study	Psychiatry	7	Eight weekly 1.5-hour mindfulness sessions, focusing on mindfulness-based stress reduction.	Empathy (yes), burnout (no), depersonalisation (no), sense of accomplishment (no)	3
Romcevich *et al*^52^	USA	Pre-post study	Medicine/ paediatrics	10	Four weekly group mind-body skills training, led by a resident with 5 years of informal meditation and mindful movement experience	Found helpful (yes), positivity (yes), stress (yes), resilience (yes), burnout (yes)	3
Zazulak *et al*^58^	Canada	Cohort study	Obstetrics and gynaecology, family medicine (GP)	35	Four weekly 3 hour art-based mindfulness sessions	Self-confidence (yes), confidence (yes), communication (yes)	4
Ireland *et al*^46^	Australia	RCT	Intern (foundation)	44	10 sessions of mindfulness education and practice adapted from validated psychological treatment programmes	Burnout (yes), stress (yes)	3
Verweij *et al*^49^	Netherlands	RCT	Medicine	148	Mindfulness-based stress reduction (MBSR) programme consisting of eight 2.5-hour sessions	Emotional exhaustion (no), sense of accomplishment (yes), worry (yes), self-compassion (yes)	2
Runyan *et al*^55^	USA	Pre-post study	Family medicine (GP)	12	Weekly 2-hour session for 4 weeks on mindfulness and self-compassion	Mindfulness (yes), empathy (yes)	4
Lases *et al*^50^	Netherlands	Cohort study	Surgery	69	Five sessions spread over 3 months on mindfulness, meditation, and self-awareness exercises delivered by athletes and managers	Found helpful (yes), stress (yes), job satisfaction (yes)	3
Rosdahl *et al*^41^	USA	Pre-post study	Family Medicine (GP), Psychiatry, Anaesthetics	30	2–3 hour mindfulness-based resilience training by clinical psychologist	Burnout (no), stress (no), mindful awareness (no)	3
Kashani *et al*^43^	USA	Pre-post study	Intensive care	21	90-min session on addressing the causes of stress, ways of framing one's mindset by attention training, relaxation techniques, meditation and mindfulness.	Found helpful (yes), burnout (no), ability to deal with stress (yes)	3
Brennan *et al*^56^	USA	Cohort study	Family medicine (GP)	23	Mindfulness and meditation classes, group reflections on individual and professional values, self-awareness, stress management, enhancing health behaviours, effective time management, and nurturing supportive relationships.	Found helpful (yes), healthy lifestyle choices (yes), resilience (yes)	4
Milstein *et al*^54^	USA	RCT	Paediatrics	15	Intervention group was trained in a psychotherapeutic technique, BATHE (Background, Affect, Trouble, Handling, and Empathy), and encouraged to use this technique three times a week for 3 months.	Found helpful (no), burnout (no)	2
Digital based, remote intervention – texts and apps							

Brazier *et al*^63^	UK	RCT	Anaesthetics	153	Twenty-two fortnightly text messages over approximately 10 months on themes including: gratitude, social support, self-efficacy, and self-compassion.	Burnout (no), wellbeing (no), sick days (no), found helpful (yes)	2
Kashat *et al*^64^	USA	Pre-post study	Surgery	8	Six weekly group meditation sessions led exclusively by the Headspace mobile application, which offers 10 min audio and video guided meditation	Mood (yes)	3
Wen *et al*^65^	USA	Pre-post study	Paediatrics	30	Open access to the Headspace application for 30 days	Mindfulness (yes), positive effect (yes), negative effect (no)	3
Improving support at work: debriefing, extra training, extra admin time							

Warren *et al*^66^	UK	Cross-sectional	Anaesthetics	40	Improving staffing levels, senior support, free parking, rest facilities, personal protective equipment supply, access to food out of hours	Found helpful (yes), felt supported (yes), felt safe (yes)	4
Stevens *et al*^67^	USA	Cohort study	Surgery	19	2 h per week of protected, non-clinical time assigned when clinical learning opportunities were lowest	Burnout (yes), wellbeing (yes), depersonalisation (no), personal accomplishment (no), QoL (no)	3
Gunasingam *et al*^69^	Australia	RCT	Mixed	31	1 h fortnightly debriefing sessions over 2 months, centring around addressing work-related stress factors, discussing coping strategies, and exploring potential avenues to enhance the wellbeing of junior medical officers.	Burnout (no)	2
Arora *et al*^68^	UK	RCT	Surgery	20	Trainees provided with guided imagery training of surgical skills	Stress (yes)	2
Ghetti *et al*^70^	USA	Pre-post study	Obstetrics and gynaecology	17	Compulsory Balint group debriefing sessions entail trainees discussing cases or situations encountered in practice that have evoked emotional responses, fostering reflection and peer support.	Burnout (no), empathy (no), confidence (yes), performance under stress (yes)	3
Yoga and exercise							

Loewenthal *et al*^59^	USA	RCT	Obstetrics and gynaecology	42	Six weekly 60 min yoga-based programme on resilience, integration, self-awareness, engagement. Online resources and home practice.	Feasibility (no), stress (yes), burnout (yes), work exhaustion (yes), interpersonal disengagement (yes).	2
Taylor *et al*^60^	Australia	RCT	Mixed	18	Eight weekly 1-hour hatha yoga sessions, and audio-guided breathing and relaxation training	Depersonalisation (yes), found helpful for mental/physical health (yes), like the exercise control group, reduced burnout.	2
Babbar *et al*^62^	USA	Pre post study	Obstetrics and gynaecology	24	8-week wellness programme consisting of yoga and nutrition classes, as well as physical exercises.	Found helpful (yes), Depersonalisation (yes), anxiety (yes),	3
Weight *et al*^61^	USA	Cohort study	Mixed	628	Team-based, 12-week, self-directed incentivised exercise programme	Quality of life (yes), burnout (no)	3
Duty work hour restriction							

Nomura *et al*^71^	Japan	Cross-sectional	Paediatrics	41	Implementation of 8-hour break between the morning round and overnight on-call shift.	Burnout (no), depression (no)	4
Parshuram *et al*^72^	Canada	RCT	Mixed	47	Random assignment to resident on-call of 24-, 16- or 12-hour duration	Sleepiness (no), burnout (no)	2
Shea *et al*^73^	USA	RCT	Medicine	103	Implementation of 5-hour protected rest period during on-call nights	Burnout (no), depression (no), empathy (no)	2
Antiel *et al*^78^	USA	Pre-post study	Surgery	156	Implementation of ACGME resident duty hour regulation	Training satisfaction (no), burnout (no), emotional exhaustion (no)	3
Brunworth *et al*^74^	USA	Cross-sectional	Surgery	295	Implementation of ACGME resident duty hour regulation (limited to max 80-hours per week)	Improvement in mental health (yes), training satisfaction (no)	4
Barrack *et al*^76^	USA	Cohort study	Surgery	55	Implementation of ACGME resident duty hour regulation	Burnout (yes), personal accomplishment (yes), emotional exhaustion (no), depersonalisation (no)	3
Martini *et al*^77^	USA	Cross-sectional	Mixed	118	Implementation of ACGME resident duty hour regulation	Burnout (yes)	4
Gopal *et al*^75^	USA	Pre-post study	Medicine	139	Implementation of ACGME resident duty hour regulation	Emotion exhaustion (yes), depersonalisation (yes), depression (yes), training satisfaction (no)	3
Goitein *et al*^79^	USA	Pre-post study	Medicine	118	Implementation of ACGME resident duty hour regulation	Emotional exhaustion (yes), career satisfaction (yes), wellbeing (yes)	3

### Mentorship and social support

Six papers, comprising cross-sectional and pre-post studies including 488 trainees, examined the impact of mentorship and social support on trainee satisfaction, burnout and professional development.^23^, ^24^, ^25^, ^26^, ^27^, ^28^ Loneliness emerged as a significant factor positively associated with burnout. This can be mitigated by friend-based and colleague-based social support, which indirectly lowers burnout by reducing loneliness.^25^ Support groups have been deemed helpful by most doctors for sharing experiences and establishing relationships, despite barriers including time constraints and group dynamics.^26^ Eisen *et al* (2013) and Webb *et al* (2015) report that peer mentorship has been highly valued, with mentees reporting increased proactivity, decision-making skills, self-confidence and a more positive professional outlook.^25^^,^^28^ Participants reported positive outcomes from peer mentorship schemes, noting improvements in behaviour, stress management, work relationships and the acquisition of transferable skills, with a substantial majority (93 %) finding the sessions beneficial.^28^ Residents with assigned faculty mentors reported higher satisfaction with their mentorship, which significantly influenced their career decisions and was deemed important to their residency experience.^23^ Dissatisfaction with emotional support from supervisors is a critical determinant of burnout among medical residents, particularly contributing to emotional exhaustion and depersonalisation.^27^

### Workshops and group discussion

Eleven studies, comprising RCTs, cohort studies, and pre-post studies including 618 trainees, examined the impact of workshops, demonstrating varying degrees of efficacy.^29^, ^30^, ^31^, ^32^, ^33^, ^34^, ^35^, ^36^, ^37^, ^38^, ^39^ Workshops covered topics including self-care techniques, self-compassion, stress management and resilience. Rich *et al* (2020) found that participants experienced a statistically significant reduction in burnout, measured by the Oldenburg Burnout Inventory, and improved boundary control 1 month post-intervention.^29^ Similarly, Saadat *et al* (2012) found that the intervention group experienced notably fewer stress-related challenges in their parenting roles and reported enhanced social support within their workplace.^32^ They also demonstrated superior problem-solving abilities when coping with stress and exhibited reduced levels of anxiety when compared to the control group or groups. In contrast, Martins *et al* (2011) noted that while there was a small reduction in alcohol use, depression and burnout at the 6-month primary endpoint, these changes did not reach statistical significance.^31^ Bragard *et al* (2009) also observed non-significant trends towards reduced burnout and preserved empathy.^38^ Despite these mixed results, qualitative feedback from participants across studies was generally positive.^29^^,^^30^^,^^34^^,^^35^

### Mindfulness

Nineteen papers, including eight RCTs, involving 1,137 residents, focused on mindfulness training as the main intervention, with mixed results.^40^, ^41^, ^42^, ^43^, ^44^, ^45^, ^46^, ^47^, ^48^, ^49^, ^50^, ^51^, ^52^, ^53^, ^54^, ^55^, ^56^, ^57^, ^58^ Mindfulness sessions across studies were delivered by various facilitators, from peers^52^ to clinical psychologists.^41^ Qualitative feedback highlighted personal and professional benefits, but while some interventions may offer subjective improvements, the objective impact on burnout and stress was inconsistent. Milstein *et al* (2009) report no significant differences in burnout symptoms between intervention and control groups, despite qualitative evidence of stress management practices among participants.^54^ Verweij *et al* (2017) found a significant decrease in perceived stress and an increase in mindful awareness post-intervention.^49^ Lebares *et al* (2021) indicated that Executive Skills Training (ESRT) could variably benefit executive function, burnout and physiological distress in trainees, although perceived stress remained unaffected.^48^ Fraiman *et al* (2022) observed that a novel mindfulness curriculum did not significantly impact emotional exhaustion, burnout, empathy or mindfulness over time.^47^ Non-RCTs also report mixed findings, with some studies including Fendel *et al* (2021) and Ireland *et al* (2017) reporting improvements in burnout, stress and empathy, while others including Rosdahl *et al* (2015) and Kashani *et al* (2015) found no significant changes or only subjective improvements.^41^^,^^43^^,^^44^^,^^46^

### Yoga and physical exercise

Four papers, ranging from CEBM evidence level 2 to 3,^22^ including 712 doctors, evaluated implementing exercise programmes.^59^, ^60^, ^61^, ^62^ Overall, these studies suggest that tailored, flexible wellness interventions can be beneficial in mitigating burnout and enhancing wellbeing among trainees. Loewenthal *et al* (2021) discovered that although in-person attendance at the yoga-based programme focusing on resilience, integration, self-awareness and engagement faced challenges due to work schedule conflicts, virtual delivery proved to be more practical.^59^ This mode of delivery resulted in sustained improvements in mindfulness, stress levels, burnout, work exhaustion, interpersonal engagement and overall physician wellbeing. Taylor *et al* (2020) reported that personalised yoga was more effective than group fitness in reducing depersonalisation and reducing burnout, though at a greater resource cost.^60^ Complementing these findings, Weight *et al* (2013) reported that participants in a team-based, incentivised exercise programme experienced higher quality of life and physical activity levels, despite no significant difference in burnout.^61^ Babbar *et al* (2019) observed significant reductions in depersonalisation, anxiety and blood pressure, as well as enhanced camaraderie and motivation, following an 8-week wellness programme.^62^

### Use of digital interventions

Three papers investigated digital interventions, including text messages and smartphone meditation applications, on resident wellbeing.^63^, ^64^, ^65^ In an RCT of 153 trainees, Brazier *et al* (2022) did not observe a statistically significant improvement in burnout or wellbeing among doctors receiving biweekly text messages on themes like gratitude, social support and self-compassion.^63^ Although there was no significant improvement in wellbeing, most participants expressed their willingness to recommend the intervention. In a similar low-cost, mass-disseminatable intervention in the form of a mindfulness app (Headspace), Kashat *et al* (2020) found reductions in negative mood, while Wen *et al* (2017) found increased mindfulness and positive effect associated with the use of a mindfulness app.^64^^,^^65^

### Enhanced workplace support

Five papers, totalling 127 participants, examined the effects of providing adequate workplace support.^66^, ^67^, ^68^, ^69^, ^70^ Warren *et al* (2021) present trainees’ narrative on the importance of having their basic physiological and safety needs addressed, such as presence of senior support, availability of rest facilities, free parking and adjustment of rota patterns, in improving wellbeing and reducing burnout.^66^ Stevens *et al* (2020) complement these findings by reporting a clinically meaningful decrease in emotional exhaustion and improvement in wellbeing with a protected, non-clinical time intervention for the purpose of learning.^67^ Arora *et al* (2011) report that providing additional training to surgical doctors through guided imagery sessions targeting surgical skills resulted in a notable reduction in both objective and subjective stress levels.^68^ Both Gunasingam *et al* (2015) and Ghetti *et al* (2009) found high levels of burnout among their subjects at baseline, which did not improve after a debriefing intervention, addressing work-related stress factors or discussing emotional cases using Balint group debriefing.^69^^,^^70^ However, the sessions were perceived positively, with a majority of participants valuing them as a source of emotional and social support.^69^ Additionally, participants reported significant improvements in confidence and ability to handle the psychological aspects of patient care.^70^

### Duty work hour restriction

Studies by Nomura *et al* (2016), Parshuram *et al* (2015) and Shea *et al* (2014), performed in Japan, Canada, and the USA respectively, all suggest that reduced work hours for medical residents may not have the straightforward positive impact on wellbeing that one might expect.^71^, ^72^, ^73^ Nomura *et al* found no statistical association between work conditions, such as the frequency of overnight calls, off-duty days and working hours, and the presence of depressive symptoms or burnout among residents.^71^ Similarly, Parshuram *et al* reported that shorter duty schedules did not significantly improve resident sleepiness or reduce somatic symptoms and burnout, as measured by the Stanford Sleepiness Scale and Maslach Burnout Inventory.^72^ Shea *et al* also found no significant differences in end-of-rotation assessments of burnout, depression or empathy between groups with and without protected sleep periods.^73^

Furthermore, six studies involving 881 residents, ranging from CEBM evidence level 3 to 4, demonstrated mixed trainee feedback on the implementation of the Accreditation Council for Graduate Medical Education (ACGME) Resident Duty-Hour Regulations.^74^, ^75^, ^76^, ^77^, ^78^, ^79^ The ACGME duty-hour regulations mandate that residents work no more than an average of 80 h per week over a 4-week period, have a maximum on-duty period of 24 h with a possible 6-hour extension for patient care continuity and educational reasons, receive one 24-hour period off per week, take in-house calls no more frequently than every third night, and have a minimum 10-hour rest period between all clinical duty periods.^80^ Gopal *et al* (2005), Barrack *et al* (2006), Martini *et al* (2006) and Goitein *et al* (2005) all report improvements in wellbeing metrics such as emotional exhaustion and burnout.^75^, ^76^, ^77^^,^^79^ In contrast, Antiel *et al* (2013) did not find a statistically significant improvement in these areas.^78^ Brunworth *et al* (2006) and Antiel *et al* (2013) expressed concerns regarding patient continuity of care and a reduction in training quality satisfaction, while Goitein *et al* (2005) reported an increase in career satisfaction.^74^^,^^78^^,^^79^

## Discussion

This systematic review examines a range of interventions from across the globe, including some conducted in the post-COVID era – a period not previously studied – targeted at enhancing the wellbeing and workplace conditions for resident doctors. The positive correlation between social support and reduced burnout underscores the need for structured mentorship programmes. These findings resonate with the theory that social connectedness is a buffer against the stressors of medical training and practice.^81^ This underscores the necessity for intervention programmes to extend beyond the residents and include training for supervisors to enhance their supportive capabilities.^28^ Workshops and group discussions have shown variable efficacy. While some studies report significant improvements in burnout, others fail to show statistical significance. This discrepancy might be attributed to methodological differences and the subjective nature of wellbeing measures. Nonetheless, the uniform positive qualitative feedback suggests that these interventions are well-received and may offer value beyond measurable outcomes, including providing a safe space for emotional expression and peer support. Mindfulness and physical exercise interventions, including yoga, provide subjective benefits in stress reduction and wellbeing. Adaptation towards virtual delivery of such programmes has been expedited by practical constraints and has demonstrated to be advantageous, suggesting a potential direction for future interventions to enhance accessibility and reduce work schedule conflicts. Although the quantitative impact on burnout is inconsistent, the qualitative benefits and improvements in related areas such as mindfulness suggest that these interventions serve as valuable elements of a holistic approach to wellbeing, which is in keeping with the findings of previously published systematic reviews.^82^^,^^83^

Mixed findings concerning duty work hour restrictions suggest that merely reducing work hours may not sufficiently address resident doctor wellbeing. While UK doctors operate under the European Working Time Directive, rather than the ACGME Resident Duty-Hour Regulations used in the USA, the issues highlighted around patient care continuity and training quality satisfaction are pertinent. These concerns suggest that there are potential compromises to be considered when balancing increased rest periods and an enhanced work–life balance, which may be universally relevant across different healthcare systems. A critical aspect to explore is whether simply reducing work hours is sufficient or whether innovative rostering strategies and bolstered support systems are key. By fostering strong relationships between supervisors and peers, as well as implementing comprehensive support mechanisms, we may mitigate the loss of continuity in patient care and maintain training quality.

Enhanced workplace support, including addressing basic physiological and safety needs as highlighted by Maslow's Hierarchy of Needs,^84^ appears to be a critical component in mitigating burnout. Interventions that offer tangible support and resources, like free parking, rest facilities and senior support, directly tackle stressors in the workplace environment. The positive reception of these measures emphasises the importance of organisational and environmental factors in wellbeing. It is interesting to note that there is a conspicuous disparity in the volume of research focusing on peripheral wellness programmes like mindfulness and yoga, compared to the paucity of studies investigating the impact of fulfilling doctors’ fundamental resource needs. It may be the case that the foundation of improved job satisfaction and effectiveness lies not in sporadic, high-visibility initiatives, but in consistently providing a robust basic working environment for doctors. This review recommends shifting research focus to study how enhancing doctors’ basic work conditions might lead to greater benefits.

Limitations of those reviews include studies largely consisting of self-selecting participants, which may introduce a bias as individuals who volunteer might already be inclined towards the intervention’s perceived benefits or be more proactive about their wellbeing. Moreover, reliance on self-reporting to gauge the effectiveness of interventions can be problematic due to cultural differences in how individuals express their wellbeing and respond to questionnaires, potentially skewing results. The quality of the intervention delivery, particularly with practices such as mindfulness, is inconsistent across studies; varying expertise and approaches of instructors can significantly impact outcomes. The duration of the interventions also varies, making it difficult to compare results or determine the optimal length of time for effective intervention. Lastly, the absence of post-exposure control groups in many studies means that long-term effects of the interventions were not measured, which limits the ability to discern the lasting impact on the participants’ wellbeing and professional lives.

Despite these limitations, urgent action is needed. While the average of OECD EU countries is 3.7 doctors for every 1,000 people, England has only 2.9, compared to 4.53 in Germany.^85^ The 2022 International Health Policy Survey of Primary Care Physicians reported that physicians in the Netherlands and Switzerland were least likely to report burnout, with both countries having a higher ratio of doctors per inhabitant.^86^ As of December 2023, the secondary care sector in England had 110,781 vacancies, including 8,758 medical positions, representing 5.7 % of total medical staff capacity. This vicious cycle stems from systemic issues, but it also exacerbates the stress, fatigue and burnout experienced by current employees. In secondary care, mental health problems have become the leading reason for sickness-related absences.^87^ Amid this challenging picture, it is concerning that NHS England recently considered reducing specialist mental health services for doctors.^88^ Physician wellbeing directly impacts the quality of patient care, the occurrence of medical errors, and the efficiency of the healthcare system.^16^^,^^89^^,^^90^ Urgent interventions are imperative to enhance the working conditions of resident doctors, as doing so is not only crucial for improving their wellbeing and quality of life, but also directly correlates with the improved patient care^91^ and workforce retention.^92^

## Conclusion

Effective interventions addressing the challenges faced by resident doctors can notably enhance their wellbeing and job satisfaction. Policymakers and healthcare administrators must prioritise the implementation and scaling of these interventions to create supportive work environments. Further research on the long-term effects and cost-effectiveness of these interventions is needed to ensure sustainable improvements in wellbeing and retention rates within the medical workforce. By fostering a supportive and holistic work environment, we can enhance the quality of patient care and ensure the sustainability of the healthcare workforce.

## Funding

None received.

## CRediT authorship contribution statement

**Yuri Hirayama:** Writing – review & editing, Writing – original draft, Validation, Project administration, Methodology, Investigation, Formal analysis, Data curation, Conceptualization. **Sunera Khan:** Writing – review & editing, Data curation. **Charn Gill:** Writing – review & editing. **Maxwell Thoburn:** Writing – review & editing. **Jennifer Hancox:** Writing – review & editing. **Jameel Muzaffar:** Writing – original draft, Supervision, Project administration, Methodology, Conceptualization.

## Declaration of competing interest

The authors declare the following financial interests/personal relationships which may be considered as potential competing interests: All authors are employees of UHB NHS FT. JM is an Educational Supervisor of doctors in training. If there are other authors, they declare that they have no known competing financial interests or personal relationships that could have appeared to influence the work reported in this paper.

## Appendix 1 Supplementary materials

Image, application 1

Image, application 2

## Footnotes

Supplementary material associated with this article can be found, in the online version, at doi:10.1016/j.fhj.2024.100195.
