# Supplementary material for: Enhancing wellbeing in medical practice: Exploring interventions and effectiveness for improving the work lives of resident (junior) doctors: A systematic review and narrative synthesis
Source: Future Healthc J. 2024 Oct 16;11(4):100195. doi: 10.1016/j.fhj.2024.100195 (PMC11584606; doi:10.1016/j.fhj.2024.100195)
Supplement: Supplementary file 1 [file mmc1.docx]

| **Introduction** | | | | | **METHOD** | | | | | | | | **Participants** | | | **Intervention and outcome** | | | | **Notes** | | |  |
| --- | --- | --- | --- | --- | --- | --- | --- | --- | --- | --- | --- | --- | --- | --- | --- | --- | --- | --- | --- | --- | --- | --- | --- |
| **Author and year** | **Title** | **Journal** | **Objective of the Study** | **Country** | **Study Design** | **Date or Year of Study** | **Total Duration of the Study** | **Study Location** | **Region** | **Population** | **Population specialty category** | **Withdrawals / Dropouts** | **Total No. of   Participants** | **Participant Inclusion and Exclusion Criteria** | **Participant age** | **Intervention** | **Duration of intervention** | **Category of intervention** | **Outcome/findings** | **Study Limitations** | **Trial Funding Source** | **Notable Conflict of Interest of Trial Authors** |  |
|  |  |  |  |  |  |  |  |  |  |  |  |  |  |  |  |  |  |  |  |  |  |  |  |
|  |  |  |  |  |  |  |  |  |  |  |  |  |  |  |  |  |  |  |  |  |  |  |  |
| Brazier et al (2022) | Dear Doctor: A randomized controlled trial of a text message intervention to reduce burnout in trainee anaesthetists | Anaesthesia | To determine if anovel text message intervention could reduce burnout and increase well-being in UK trainee anaesthetists | UK | Randomized control trial | 2019 | 11 months | UK | National, mostly London | Anaesthetic trainees | Anaesthetics | 494 did not continue after finishing baseline survey | 153 | Core Training Year 2 (CT2), orSpecialty Training Years 3 or 4 (ST3 or ST4) of the UK training programme | Not stated | All received one initial message sharing support resources. Intervention group received 22 fortnightly text messages over approximately 10 months. Messages drew on 11 evidence-based themes including: gratitude; social support; self-efficacy; and self-compassion.  (Two-group non-blinded randomised controlled trial) | 10 months | Digital or mobile health (mHealth) intervention | Primary outcomes were burnout (Copenhagen Burnout Inventory) and well-being (Short Warwick-Edinburgh Mental Well-being Scale) measured via online survey at the end of the intervention.Secondary outcomes: meaning in work; professional value; sickness absence; andconsideration of career break. Included post-hoc, was the impact of COVID-19 (thefirst UK wave of whichcoincided with the second half of the trial). No significant group differences in: burnout (p=0.45); well-being (p=0.40); meaning (p=0.77);value (p=0.99); sick days (p=0.56); or consideration of career break (p=0.24). In the exploratory moderation analysis, the intervention was associated with significantly different burnout levels in participants who experienced difficulties (b=-9.56, 95% CI-17.35 to-1.77, p=0.02) or reported COVID-19 had a big negative impact (b=-10.38, 95% CI-20.57 to-0.19, p=0.05). Those who experienced difficulties were more likely to have reported a large negative impact from COVID-19 (Spearman correlationq=0.38;v2(1)=21.8, p<0.001). A small number of the final intervention group participants (11%; n=8) said they would not recommend the intervention and 23% (n=17) said they were not sure. Despite no overall impact on outcomes, the majority of participants said they would recommend the intervention. | Self-selection happened during the recruitment stage (although not during the trial period): those with higher burnout at baseline were less likely to participate in the trial.   The trial was not powered to detect if interventioneffects were moderated by baseline burnout levels (Existing literature indicates that baseline burnout level moderates intervention effect size).   in the pandemic context, the number of support resources available to clinicians and signposting tothese resources was increased. Could this have decreasedthe impact of the text message intervention, given well-being support was being provided by other channels,leading to the null result observed overall? | NIHR | None |  |
| Rich et al (2020) | Evaluation of a novel intervention to reduce burnout in doctors-in-training usingself-care and digital wellbeing strategies: amixed-methods pilot | BMC Medical Education | To assess thefeasibility, acceptability and impact of a novel intervention to reduce burnout and improve wellbeing. | UK | Pre post study | Not stated | 2 months | UK | London | NHS post-graduate medical trainee | Medicine | 34 did not attend workshops | 22 | NHS post-graduate medical trainee | Not stated | Workshops (+ group discussion, experiential and reflective exercises) facilitated by Health Psychologist and Human-Computer Interaction Specialist. 1. Advice on selfcare techniques (e.g., physical activity, eating habits, sleep strategies), self-compassion and mindfulness meditation. 2. Digital wellbeing advice on taking control of technology use through microboundaries to improve work-life balance. The microboundary strategies included: email management; notification and awareness cues management (e.g., disabling “read receipts” and “online status” in the social messaging app “WhatsApp”), and expectation of availability management (e.g., setting an out of office message for the weekends). More details about these strategies are available on the iWARDS website (https://iwards.wordpress.com/) | Six two-hour workshops | Workshop and group discussion | The intervention was well-received, with all trainees finding the workshop useful and saying they would recommend it to others. At baseline most participants had scores indicative of burnout on both the disengagement (82%) and exhaustion (82%) subscales of the Oldenburg Burnout Inventory.  One month postintervention, participants had a statistically significant reduction in burnout (Oldenburg Burnout Inventory)n(both disengagement and exhaustion) and improvement in boundary control ( boundary control sub-scale of the Work-Life Indicator) . Wellbeing scores (Warwick–Edinburgh Mental WellBeing Scale) also improved, but differences were not statistically significant.  Qualitative analysis indicated participants had welcomed a safe space to discuss stressors and many had implemented digital wellbeing strategies to manage their smartphone technology, and increased self-care such as mindfulness practice and walking in green space. Thematic analysis revealed the following seven themes: 1) Opportunity for reflection and to prioritise wellbeing 2) The value of sharing and hearing the experiences of others 3) Workshop content 4) Self-care goals and outcomes; enablers and barriers to implementation 5) Microboundaries goals and outcomes; enablers and barriers to implementation 6) Future intentions7) Improvements for future workshops | Lack of a control group, meaning we cannot exclude other possible confounding factors. C hallenges with attendance, with only 51% of those signing up actually attending the sessions. Small sample size limits the generalisability of the findings and could have resulted in the lack of a detected effect for improvement in mental wellbeing scores, which were not statistically significant (type II error). Participants were a selfselecting sample. | Grand Challenge Grant from UCL | Dr. Antonia Rich and Professor Anna Cox were awarded a Grand Challengegrant from UCL |  |
| O’Riordan et al (2020) | Interventions to improve wellbeing among obstetriciansand midwives at Cork University Maternity Hospital | Irish Journal of Medical Science | To investigate whether an intervention which increases support for staff is feasible to implement and effective at improvingstaff wellbeing | Ireland | Pre post study | 2016-2017 | 13 months | Ireland | Cork | Midwives and obstetrics and gynaecology doctorsin training | O&G | 10/18 dropout for doctors in training | 8 | Doctors in training (O&G), mid-wives | Median age 31(range = 25 to 53) | Pocket card and posters promoting self-care and resilience; team bonding sessions for the doctors in training | One session. | Debrief, Reminder posters | The DIT team bonding sessions were discontinued after onesession. Further attempts were made to organize subsequent sessions but there was low interest among the DITs. Amongst the MBI’s Burnout, ProQoL’s CompassionFatigue, and Perceived Stress Scale, only reported burnout score differed significantly (p<0.05) pre- and post-intervention.  Feedback on head hygiene posters:  "Made you think to mind yourself" "Gives you time to reflect and think of what you need to do to benefit your mental health" "Permission to think about your thoughts and feeling. A regular reminder to do so" "The 5 moments of head hygiene poster was a handy reminder to take a little break each day and mind my emotional wellbeing" "A gentle reminder of ways to cope with stressful situations" "Makes you think to care for yourself and co-workers. To be more mindful of how you are feeling and what those around you might be going through if they have had a tough day" Confusement: "To be honest I did not realise they were not hand hygiene posters until it was pointed out!" "I found it confusing at first–as they seemed about hand hygiene" "Not initially very clear on what it was" "I personally found it confusing–I am not a visual learner" | High dropout rate. Amaglamation of scores from midwives and doctors despite the two groups undergoing different interventions. | Irish Centre forFetal and Neonatal Translational Research and from the ScienceFoundation Ireland in part (12/RC/2272) | Not stated |  |
| Bu et al (2019) | Mindfulness intervention for foundationyear doctors: a feasibility study | Pilot and Feasibility Studies | To assess the feasibility of conducting a full-scale evaluationof a mindfulness intervention among UK foundation doctors to reduce stress and burnout | UK | Pre post study | 2017 | 3 months (Sep-Dec) | UK | Liverpool | F1 and F2 doctors | Intern/Foundation | 20 | 10 F1s and 10 F2s. Of these 20, 10 were fe-male and 10 were male | (a) being a foundation junior doctor inthe hospital and (b) being able to participate in at leastfour of the six course sessions | Not stated | a 6-week mindfulness course (six 2-h sessions) which wasdelivered by Breathwork (operate in accordance with the UKGood Practice Guidelines for mindfulness teachers) | 6 weeks | Mindfulness | The primary outcome measure was self-reported stresslevels measured immediately before and after the course (A one-item self-reported Likert scale). Median pre 6.5 (range = 2 to 9) --> median post 5.0 (range = 2 to 8) p= .04. Secondary outcome: subjective experiences ofdoctors participating in the mindfulness course with regard to four domains including self-reported mindfulness, its impact on their wellbeing, impact on workinglife and impact on relationships with patients. All participants reported that they were more mindfuland had improved overall wellbeing. In terms of overallwellbeing, doctors mentioned the course made themrealise it is‘okay to take time out for self’,‘reduced anx-iety’, and‘encouraged me to take time out of the day andcheck in with my own sense of wellbeing’. Another re-ported‘relaxation techniques at home have really helpedme switch off and sleep’, and one doctor stated thecourse had a‘positive impact on my thought process’.Twelve out of 14 participants reported that the mindful-ness course had a positive effect on their working lifeand their relationship with patients, whereas two partici-pants reported no change on these aspects. Regardingworking life, doctors reported that they find it easier to‘prioritise jobs’and‘concentrate better’, and they felt theywere‘more of a pleasure to work with’. For their rela-tionships with patients, they also reported they were‘more understanding of difficult patients’,‘more mindfulof what they are going through’,‘more patient’, and‘morecompassionate’. | major limitation was the use ofnon-validated brief measures of stress and wellbeing. the sample size was small. limited demo-graphic information. No follow-up to assess maintenance. lack of controlgroup and randomisation process | Royal Liverpool and Broadgreen UniversityHospital Trust Medical Education Department. | None |  |
| Warren et al (2021) | Trainee doctors’ experiences of learning and well-being while working in intensive care during the COVID-19 pandemic: a qualitative study using appreciative inquiry | BMJ Open | Aimed to understand the experiences of trainees working in a large intensive care unit during the first surge of the COVID-19 pandemic from an educational and operational perspective in order to highlight what worked and what could be improved | UK | Cross-sectional study | 2020 | 1 month (Jul) | UK | Birmingham | All trainees in anaesthesia and intensive care working on the intensive care unit during the first surge of COVID-19 pandemic | Anaesthetics | None | 40 | Inclusion: all non-consultant doctors. Exclusion: consultants, shielding doctors, maternity leave | Not stated | N/A | N/A | Improving resources | A qualitative study using peer-to-peer semistructured interviews. Four over-arching themes were identified. These were: feeling safe and supported (24/7 consultant presence, sufficient staffing levels); physical demands; the emotional burden of caring; and a sense of fulfilment, value and personal development. Positive aspects of the organisational response to the pandemic included food, free parking, rest facilitie, personal protective equipment supply, team working and well-being support (peer support team). These themes describe the positive and negative aspect of working during the pandemic and can be seen as aligning with Maslow’s hierarchy of human needs: basic needs (physiological and safety), psychological needs (belonging and self-esteem) and self-fulfilment (self-actualisation). For each grade of respondent, the most frequent suggestions for improvement were around addressing basic needs (physiological and safety) such as through improved rota patterns and rest facilities; fewer suggestions were made that related to higher needs Suggestions for improvement focused on basic needs e.g. rest facilities, rota patterns and hierarchies, creating opportunities for reflection (formal debriefing sessions) and ensuring continued educational and training opportunities despite operational demands. Teaching (‘I missed formal teaching and the contact with a senior and a focused coming together of a speciality group'). Opportunities to observe communication around decision making for significant events like withdrawal of life-sustaining therapy. This led to emotional distress with one junior trainee noting that, ‘the decisions were not always well explained (to the trainees)’ | Only one interviewer, not recorded. Not everyone working on the COVID-19 rota was inter-viewed and also trainees who were struggling the most were excluded, which may have introduced a source of bias. The study was conducted in a single trust, and experiences may be different in different settings. | None | None |  |
| Hsu et al. 2010 | Mentorship in Otolaryngology Residency:The Resident Perspective | The Laryngoscope | To assess the currentstate of mentorship in otolaryngology residency. | USA | Cross-sectional study | 2009 | 2-week period beginning May 12, 2009 and ending May 25,2009 | USA | New York | Chief residents in ENT | Surgery | None | 47 | Residents of accredited otolaryngology residency programs in the United States | 32 years (standard deviation, 2.4 years) | N/A | N/A | Mentorship | Statistically significant higher scores for satisfaction with mentorship received during residency training for individuals with assigned faculty mentors versus those without (median, 4 vs. 3; mean, 4.1 vs.3.5;p<.05). 'Mentorship influenced my career decisions, Mentorship was important to my residency experience' | Poorly written, making interpretation of finding difficult and limited. | None | None |  |
| Stevens et al (2020) | Association of Weekly Protected Nonclinical Time With Resident Physician Burnout and Well-being | JAMA Otolaryngol Head Neck Surg | To design a departmental-level burnout intervention, evaluate its association with otolaryngology residents’ burnout and well-being, and describe how residents used and perceived the study intervention | USA | Cohort study | 2017-2018 | 9 months | USA | Minnesota | All resident physicians (PGY1-PGY5) in the Department of Otolaryngology | Surgery | None stated | 19 | Inclusion: All resident physicians (PGY1-PGY5) in the Department of Otolaryngology. Exclusion: Principal investigator | Not stated | Prospective,non randomized crossover study: 2 hours per week of protected, nonclinical time assigned when clinical learning opportunities were lowest, in the intervention phase of the study for the first or last 6 weeks of a quarterly rotation. They were encouraged to use this time in a way they felt would decrease their own personal burnout and increase their well-being, whether performing work-related administrative duties previously performed on personal time or fulfilling obligations that are integral to personal health and well-being that can only be completed during normal business hours. | 32 weeks | Protected administration time | Burnout was measured primarily by the Maslach Burnout Index (MBI) and secondarily by the Mini-Z Survey (Mini-Z). Well-being was measured primarily by the Resident and Fellow Well-Being Index (WBI) and secondarily by a quality-of-life (QOL) single-item self-assessment (SA). Among the 19 residents in the study (10 men [53%]), the overall protected time intervention (week 0 to week 32) was associated with a mean decrease of 0.63 points (95% CI, −1.03 to −0.22 points) in the Maslach Burnout Inventory emotional exhaustion score, indicating a clinically meaningful decrease in burnout, and a mean decrease of 1.26 points (95% CI, −2.18 to −0.34 points) in the Resident and Fellow Well-Being Index score, indicating a clinically meaningful improvement in well-being. The baseline to week 32 mean changes in the Maslach Burnout Inventory depersonalization score, Maslach Burnout Inventory personal accomplishment score, and quality-of-life single-item self-assessment were not clinically meaningful. | Study was conducted solely among otolaryngology residents in 1 department. There was also a large number of surveys, which could reasonably lead to survey fatigue over time. The nonrandomized assignment of intervention periods is also a limitation in that it prevents estimation of order and carryover effects. Other sources of potential bias include the lack of blinding, observer bias, cause-effect bias, secondary gain bias, the Hawthorne effect, and the Dunning-Kruger effect. | Minnesota Lions Foundation. National Institutes of Health’s National Center for Advancing Translational Sciences | Stevens - grants from the Minnesota Lions Foundation. Ms. Davey is affiliated with the University of Minnesota Biostatistical Design and Analysis Center (part of the Clinical Translational and Science Institute). Clinical Translational and Science Institute received a Clinical and Translational Science Award. Dr. Lassig- grants from Lions Foundation during the conduct of the study. |  |
| Brunworth et al (2006) | Impact of Duty Hour Restrictions on Otolaryngology Training: DivergentResident and Faculty Perspectives | The Laryngoscope | To examine the impact of implementation of working hours' restrictions on otolaryngology programs and to explore their perspectives | USA | Cross-sectional study | Not stated | Not stated | USA | San Diego, California | ACGME-accredited otolaryngology training program residents | Surgery | None stated | 295 | ACGME-accredited otolaryngology training program residents | Not stated | Summary of Accreditation Council for Graduate Medical Education Resident Duty-Hour Regulations:  Residents are limited to a maximum of 80 duty hours per week, including in-house call, averaged over 4 weeks. Residents must be given 1 day of 7 free from all clinical and educational responsibilities, averaged over 4 weeks. Residents cannot be scheduled for in-house call more than once every 3 nights, averaged over 4 weeks. Duty periods cannot last for more than 24 hours, although residents may remain on duty for 6 additional hours to transfer patients, maintain continuity of care, or participate in educational activities. Residents should be given at least 10 hours for rest and personal activities between daily duty periods and after in-house call. In-house moonlighting counts toward the weekly limit. | N/A | Duty hour restriction | Participant responses indicated that the majority of training programs (65%) had implemented at least one major infrastructural change specifically to comply with ACGME duty-hour restrictions. Strategies included: tracking resident work hours electronically (35.7%), utilization of “home call” for first-call residents (33.1%), and hiring additional healthcare professionals (nurses, physician assistants, and so on) (23.1%). Most agreed that resident mental health had indeed improved (67%). Only 34.8% of participants felt that residents would have a more fulfilling training experience as a result of limited work hours, whereas the majority (52.2%) disagreed. In fact, 46.3% of those surveyed believed that the regulations have had a negative effect on the resident training experience.When asked if the restrictions have had a negative impact on patient care, the majority (61%) of all respondents answered “no,” but a surprising 33% answered “yes.” | ?how applicable are these findings if not all programmes have implemented consistent infrastructural changes. Self selecting sample. US residents and written in 2006, ?applicability to UK trainees currently | Not stated | Not stated |  |
| Loewenthal et al (2021) | Evaluation of a Yoga-Based Mind-BodyIntervention for Resident Physicians:A Randomized Clinical Trial | Global Advances in Health and Medicine | To assess the feasibility of a yoga-based Mind-body interventions (MBIs) called RISE (resilience, integration, self-awareness, engagement) for residentsamong multiple specialties and academic medical centers | USA | Randomized control trial | 2018 and 2019 | Not specified | USA | Massachusetts | Residents of Brigham and Women’s Hospital, BethIsrael Deaconess Medical Center, Boston Children’sHospital, and Harvard combined residency programs | O&G | 16 (clear explanation: lost to f/u, withdrawal) | Intervention 26, control 16 | Inclusions: residents. Exclusions: Fellows, residents that have practiced a mind-body technique for more than 25 hours inthe past six months | Intervention mean 29.3y (range 26–33)  Control 29.1y (range 27–33) | RISE (resilience, integration, self-awareness, engagement) is a program of yoga-based practices, developed by Kripalu Center for Yoga & Health.  Sessions contained a combination of didactic and experiential mate-rial. Participants had access to online resources thatreinforced content and were asked to maintain a dailyhome practice.  The waitlist control group received onesession at the end of the study period, which included anoverview of didactic material and practices | 60-minute weekly sessions over a six-week time period | Yoga and exercise | Feasibility was assessed according to six dimensionsas described in recommendations for the design of feasibility studies from the National Cancer Institute,utilized in other studies of mind-body interventions: 1)demand,2)implementation,3) practicality,4)acceptability,5)adaptation, and 6)integration.  Feasibility of in-person attendance was rated as 28.9 (SD 25.6) on a 100-point visual analogue scale. Participants rated feasibility as 69.2 (SD26.0) if the program was offered virtually. Therefore, RISE was not rated as highly feasible by residents.Most importantly, residents reported that their workschedule conflicted with the delivery of the program. Most participants utilized online RISE resourceseven if they did not attend in-person sessions, suggestingvirtual delivery as a potential adaptationHowever, those who received RISE reported improvements in mindfulness, stress, burnout, work exhaustion, interpersonal disengagement, total burnout and physician well-being from baseline to post-program, which were sustained at two-month follow-up (p<0.05). | Conducted in aself-selected, single geographic area, lower surgical resident participation, limiting generalizability. Did not explore impact of MBI post 2-months | Osher Pilot Research Award (SBSK) from the OsherCenter for Integrative Medicine, Brigham and Women’sHospital, Harvard Medical School; Partners Centers ofExpertise in Medical Education grant, Partners HealthCareGraduate Medical Education (JL) | None |  |
| Rogers et al (2016) | Getting by with a little help from friends and colleaguesTesting how residents’ social support networks affect loneliness and burnout | Canadian Family Physician | To determine how residents’ relationships with their sources of social support (ie, family, friends, and colleagues) affect levels of burnout and loneliness | Canada | Cross-sectional study | 2014 | Not stated | Canada | Vancouver | Physician-trainees in the university’s postgraduate medical education program | Medicine | 36 | 198 (gave full response) | Residents in the Faculty of Medicine’s Postgraduate Medical Education Program at the University of Brit-ish Columbia in Vancouve | Not stated | N/A | N/A | Social support | higher levels of friend-based support are significantly associated with a lower work-related burnout score (b=-0.062; P<.05). However, once loneliness is controlled for this initial association for friend-based support is attenuated and is no longer statistically significant. Seemingly unrelated regression analyses indicated that loneliness was significantly (P<.01) and positively associated with both personal and work-related burnout scores. Greater friend-based and colleague-based social support were both indirectly associated with lower personal and work-related burnout scores through their negative associations with loneliness. Social relationships might help residents mitigate the deleterious effects of burnout. By promoting interventions that stabilize and nurture social relationships, hospitals and universities can potentially help promote resident resilience and well-being and, in turn, improve patient care | Lack of sample heterogeneity (sample consisted of residents from one Canadian university and province). Response rate could indicate a possible systematic nonresponse bias, whereby residents with either low or high levels of burnout chose to not com-plete the survey | Not stated | None |  |
| Axisa et al (2019) | Burnout and distress in Australian physician trainees: Evaluation of a wellbeing workshop | Australian Psychiatry | To evaluate the effectiveness of a workshop intervention to promote wellbeing for Australian physician trainees using a randomized-controlled design | Australia | Randomized control trial | 2014 | 10 months | Australia | New South Wales | Adult Internal Medicine and Paediatrics and Child Health training divisions of the Royal Australasian College of Physicians | Medicine, Paediatrics | 18 (did not attend all workshops or lost to f/u) | 59 (control 25, intervention 34) | Physician trainees completing their RACP basic physician training in New South Wales (NSW) hospitals | 25-44y (mean not available) | 1 workshop - Following review of the international literature, a work-shop was developed in consultation with local experts: strategies for wellbeing and stress management and to encourage participants to apply these strategies in their own lives. Topics in the workshop included stressors relating to work–life balance, understanding wellbeing and resilience, mindfulness, barriers to looking after wellbe-ing, giving and receiving feedback and stress manage-ment strategies | workshop duration was 4.5 hours including a 30-minute meal break where a catered meal was provided | Workshop and group discussion | Workshop evaluations were very positive. Most respond-ents (83%) were able to utilize some of the workshop strategies for 12 months post workshop.There was a small reduction in alcohol use, depression and burnout in the intervention group compared with the control group measured at the primary end-point at 6 months, but these changes did not reach statistical significance. | Sample size is small despite a large potential study cohort and extensive recruitment advertising. Lack of control over work rosters, difficulty swapping shifts, being on call or studying for the RACP exams. | None | None |  |
| Goldhagen et al (2015) | Stress and burnout in residents: impact of mindfulness-based resilience training | Advances in Medical Education and Practice | To test the hypothesis that a mindfulness-based resilience intervention would decrease stress and burnout in residents | USA | Pre post study | 2012 and 2013 | Not specified | USA | North Carolina | Resident physicians from the Departments of Family Medicine, Psychiatry, and Anesthesia at Duke University | Family Medicine (GP), Psychiatry, Anaesthetics | 17 participants did not finish final survey (47 patients initially recruited) | 30 | Residents at residency programs with program directors’ interest in offering their residents the mindfulness-based resilience training | 20-39 | Two or three 1-hour mindfulness-based resilience training sessions. These group sessions were developed and presented by a clinical psychologist with expertise in mindfulness-based tech-niques | 2-3h | Mindfulness | Surveys were distributed just prior to the first session and just after the last session (2–8 weeks apart, based on program scheduling) Analysis of the surveys before and after the intervention showed no significant short-term change in stress, burnout, mindful-awareness, or cognitive failure. There was a trend for females and post-medical school graduate year 1 and 2 (PGY1 and PGY2) residents to have a reduction in DASS-21 scores after intervention. There was also a trend of reduced stress and burnout in residents who perceived higher stress. |  |  |  |  |
| Eisen et al (2013) | Peer mentoring: evaluation of a novel programmein paediatrics | Archives of disease in childhood | To assess demandand need for peer mentoring and to explore the benefitsfor both peer mentees and mentors | UK | Pre post study | Not stated | Not specified | UK | London | Postgraduate trainees in paediatrics | Paediatrics | None | 18 | first-year postgraduate traineesin paediatrics | Not stated | Peer mentors undertook a 3-day coaching and mentoring course using an established model of coaching. Mentorship provided for 1 year. | 1 year | Mentorship | Ninety-four per cent of mentees felt their peermentor had been a significant source of support. The controlgroup, without a peer mentor, also valued support of trainee col-leagues and friends/family (both 94%; 24/26), but 81% (21/26)‘wished they had been allocated’a mentor. Seventy-eight per cent (14/18) of peer mentees reportedbeing more proactive in seeking learning opportunities, improved decision-making skills, stress management, benefits included improved relationships with colleagues, clinicaland examination performance and work–life balance. Peer mentees also reported changes in outlook. Eighty-nineper cent (16/18) reported improved self-confidence.Eighty-three per cent (15/18) felt a greater sense of realism:“Ifeel reassured that my concerns are normal for my stage”.Seventy-eight per cent (14/18) reported a positive change intheir professional outlook. | Non standardised contact between mentor and mentee - hence study acts as observational, rather than a cohort study. Self-selected sample. | Faculty DevelopmentDepartment, London Deanery | None |  |
| Cheung et al (2020) | Preliminary Efficacy of a Brief Mindfulness Interventionfor Procedural Stress in Medical InternSimulated Performance:A Randomized Controlled Pilot Trial | The Journal of Alternative and complementary medicin | To examine the efficacy of bolstering an established Simulation-based mastery learning (SBML) program for medical residents with a brief mindfulness intervention (called a PITSTOP) to reduce procedural stress and improve simulator performance | USA | Randomized control trial | June 2015 to January 2018 | Not specified | USA | Chicago | Trainee doctors | Medicine | 7 | Intervention 13, control 13 (total 26) | Postgraduate year (PGY) 1 in-ternal medicine residents at Northwestern University Fein-berg School of Medicine. Exclusion: doctors who practice regular (>3 times a week)mindfulness meditation for the past year or were enrolled inother studies using psychologic interventions | 28.50 (SD 2.45 | The authors developed a brief (12-min) videoto teach foundational principles of mindfulness, which cul-minated in the instruction of the PITSTOP technique  (Partially blinded, parallel-group, randomized, repeated-measures intention-to-treat design) | 12 min | Mindfulness | Primary outcom: comparison of each group’s simulator performance duringpre- and post-tests. Secondary outcomes: changes in groups’ procedural stress during these tests (assessedusing self-reported, instructor-rated, and physiologic indicators). Residents who watched the PITSTOP video (ause; Inhale; Take note of yourSelf and the Task at hand; Observe the experience withoutjudgment and/or where the mind may have wandered andgently bring it back; and finally, Proceed with the task oractivity when ready) before their SBML training made fewer procedural errors relative to controls during their pretest for intrajugular CVC insertion (p=0.03). PITSTOP participantsalso had lower heart rate (p=0.03) and less visible trembling (p=0.003) relative to controls at the post-test. Authors conclude that brief mindfulness training such as thePITSTOP offers a simple, scalable adjuvant to improve self-regulation, and to potentially enhance and reinforce medical learning and performance. | study was performed at a single institution, with a smallnumber of participants, and a 27% attrition rate | None | None |  |
| Arora et al (2011) | Mental Practice: Effective Stress Management Training for Novice Surgeons | American College of Surgeons | To investigate if Mental Practice (MP) reduces stress in novice surgeons | UK | Randomized control trial | February 2008 to April 2008 | 2 months | UK | London | Trainee surgeons | Surgery | 2 | Intervention 10, control 10 (total 20) | Inclusion: if they had never performed any surgical procedure previously, but did have theopportunity to assist others in the OR | Intervention 22 (21–23), Control 22 (20–23) | A validated mental practice training strategy. Participants watched a video of anexpert surgeon performing a live lapchole to gain a mental representation of the task. They practiced guided imagery with a facilitator using an MP “script.” which contained a sequence of procedural steps for thetask but, inaddition, a set of detailed and vivid im-agery cues designed to enrich participants’ mental representation of the skill being learned. | 30 min of MP video | Improving resources | Comparing the MP group with controls, subjective stress (STAI) was lower for the MP group (median 8.40 vs 11.31, p<0.01). Objective stress was alsosignificantly reduced for the MP group in terms of the average HR (median 72 vs 88 beats/minute, p0.0001), maximum HR (median 102 vs 119 beats/minute, p<0.01), and cortisol(median 2.26 vs 3.85 nmol/L, p<0.05). Significant negative correlations were obtainedbetween stress and imagery, indicating that improved imagery was associated with lower stress(p0.05) | the procedure was conducted on a simulatorand with participants in a single institution | The BUPA Foundation and the Association ofSurgical Education CESERT grant, NIHR | None |  |
| Taylor et al (2020) | Personalised yoga for burnout and traumatic stress in junior doctors | Postgrad Med J 20 | To assess the feasibility, acceptability and effectiveness of mindfulness-based and fitness interventions in junior doctors. | Australia | Randomized control trial | November 2018 and April 2019. | 5 months | Australia | Sydney | Junior doctors at at the Royal Prince Alfred Hospital | Mixed | 3 | 18 | Junior doctors at at the Royal Prince Alfred Hospital | 30±4 (23 to 37) | Hatha yoga consisting of eight, 1-hour private yoga sessions, once a week. 4-hour workshop/retreat, two eHealth video classes, and audio-guided breathing and relaxation was also provided | 8 weeks (8h) | Yoga and exercise | The findings support individual-directed or physician-directed interventions in a wellness programme for medical doctors. Personalised yoga significantly reduced depersonalisation (z=−1.99, p=0.05) compared with group fitness on the Maslach Burnout Inventory (MBI-HSS (MP)) and showed greater flexibility changes. Both interventions reduced burnout. Participants doing one-to-one yoga rated it more highly overall (p=0.02) than group fitness, and reported it comparatively more beneficial for mental (p=0.01) and physical health (p=0.05). One-to-one yoga was better adhered than fitness, but was more resource intensive | small sample. Female>Male participants | The Sydney Local Health District and the NSW Ministry of Health JMO Be Well Program fund the MDOK program delivering the fitness control arm | BR is the Chief Medical Wellness Officer, WellMD Centre, Sydney Local Health District. JT worked separately to the trial as a group yoga and meditation instructor on the MDOK programme. |  |
| Gunasingam et al (2015) | Reducing stress and burnout in junior doctors:the impact of debriefing sessions | Postgrad Med J | To examine the prevalence ofburnout in a cohort of junior doctors and whetherdebriefing sessions reduced levels of burnout | Australia | Randomized control trial | Not stated | Not specified | Australia | New SouthWales | Postgraduate year 1 doctors ina single hospital was undertaken during a rotation termin 2011. | Mixed | None stated | 31 in total (13 intervention, 18 control) | Postgraduate year 1 doctors ina single hospital was undertaken during a rotation termin 2011. | 20-30y (median not available) | Randomly assigned to a group who were to receive four 1h fourtnightly debriefing sessions over 2 months, or, to the controlgroup, who had no debriefing session. Focus group after debriefing session:explored themes around work-related stressors, coping mechan-isms and potential strategies to improve Junior Medical officer well-being. | 2 months | Debrief | Burnout is prevalent among postgraduate year 1 doctors, and they value the emotional and social support from attending debriefing sessions. At baseline, 21/31 (68%) participants displayed evidence of burnout in at least one domain as measured by the Maslach Burnout Inventory. Burnout was significantly higher in women. There was no significant difference in burnout scores with debriefing. The intervention was well received with 11/18 (61%) suggesting they would recommend the strategy to future junior doctors and 16/18 (89%) found that the sessions were a source of emotional and social support. Identified stressors elicited during the focus groups included difficulty in maintaining work–life balance, lack of support by senior staff and uncertainties in relation to career paths and training. Identified coping mechanisms included socialising, alcohol, informal and formal debriefings with colleagues, and formal support systems from hospital teams. | No statistical analysis of difference of demographic info of intervention vs control groups. Study waslikely underpowered to detect a difference in burnout follow-ing debriefing. Potential fors election bias with those who did not elect to participate. Attendanceat the debriefing sessions was not always 100% | Not stated | None |  |
| Kashani et al (2015) | Stress and burnout among critical care fellows:preliminary evaluation of an educational intervention | Medical Education Online | To assess levels of burnout, perceived stress, and quality of life in critical care fellows, and thei mpact of a brief stress management training on these outcomes | USA | Pre post study | 2013 | Not stated | USA | Minnesota | Critical care fellows | Intensive Care | 3 | 21 | all adult critical care fellows enrolled in the Pul-monary and Critical Care Medicine, CriticalCare Internal Medicine, Critical Care Anesthesia,and Neurology Critical Care training fellowshipprograms at Mayo Clinic (Rochester, MN) | 25-50 (mode 32) | 90-min stress managementintervention adapted from the previously-piloted Stress Management and Resi-liency Training (SMART) program at Mayo Clinic. Theprogram consisted of a presentation and handouts addressing the causes of stress, and introduced ways offraming one’s mindset by attention training (i.e., focusingon the novel and shifting one’s attention from inward tooutward). This was followed by structured relaxation training by utilizing paced breathing medita-tion and deep diaphragmatic breathing. | 90 min | Mindfulness, resilience training | Among the intervention cohort, burnout did not significantly improve (as measured by mean satisfaction with life scale, or Maslach Burnout Inventory (MBI). Despite no documented improvementsin fellows’ self-reported levels of burnout, 67% expressed a strengthened ability to deal with stressful situations, and 61% reported using the techniques after 1 year. Fellows who participated in the intervention rated the effectiveness of the course as 4 using the 5-point Likert scale | Small sample size and singular study setting reduce therigor and generalizability of our findings. Lack of control group. Although fellows were assured that their responses were confidential; it is unclear how free they felt to share their opinions un-reservedly and honestly | None | None |  |
| Fendel et al (2021) | The impact of a tailored mindfulness-based programfor resident physicians on distress and the quality ofcare: A randomised controlled trial | Journal of Internal Medicine | To examine the impact of a tailored mindfulness-based program (MBP) for resident physicians on distress and the quality of care | Germany | Randomized control trial | September 2018 to May 2020 | 32 months | Germany | Freiburg | Physician in training | Medicine | None stated | Intervention(n=76; Control(n=71) | physicians younger than 45, with an ongoing position as a resident physician at base-line, minimum employment of 40% | 31.02 y (SD 3.43) | The interventiongroup took part in an 8-week (135 min one evening per week), tailored MBP that included a coursebook. Delivered by three psychiatrists who are highly experienced mindfulness instructors. The MBP was followed bya 4-month maintenance phase. The active control group received the coursebook for self-study. introduced mindfulness as a practice ofself-care, in order to promote personal well-being, meaning and professional fulfilment rather than as a means to foster stress resistance. Control group received the same coursebook on the same weekly basis as the intervention group, except that the coursebook for the control group did not contain and a description of practical exercises. | 8 weeks | Mindfulness | The intervention group showed greater improvements in burnout at 2 months,d=0.32,p=0.046,in perceived stress (d=0.31,p=0.046) and perceived job strain(d=0.33,p=0.026) at 2 months,and in supervisor rated empathy (d=0.71,p=0.037) and colleague rated attentiveness (d=0.85,p=.006) at 6 months.There was no difference between groups in depression, anxiety, hair cortisol secretion, self-reported medical errors and third-party ratings by patients. | Linear mixed-effects modeling revealed differences at T2 and T3 in perceived stress and in mindfulness.   **Burnout at 2 and T3 showed similar increase in both groups.** Cognitive control S25:U25decreased more in the control arm at T2 and at T3 | German Research Foundation, the Collaborative Research Center 1015 Otium. Boundaries, Chronotopes, Practices for funding. | None |  |
| Lebares et al (2019) | Efficacy of Mindfulness-Based Cognitive Training in Surgery Additional Analysis of the Mindful Surgeon Pilot Randomized Clinical Trial | JAMA Network Open | To explore potential benefits to stress, cognition, and performance in postgraduate year1 (PGY-1) surgery residents receiving modified mindfulness-based stress reduction (modMBSR) | USA | Randomized control trial | 2016 to 2017 | 18 months | USA | California | PGY-1 surgery residents | Surgery | 2 dropout | 10 intervention, 9 control | Postgraduate year 1 (PGY-1) surgery residents at UCSF, without a current mindfulness meditation practice | Intervention 29.0 (2.4), control 27.4 (2.1) | Weekly 2-hour modified mindfulness-based stress reduction (modMBSR) classes and 20 minutes of daily home practice duringan 8-week period vs an active control (different content, same structure) | 8 weeks | Mindfulness | Linear mixed-effects modeling revealed differences at T2 and T3 in perceived stress and in mindfulness. Working memory increased more at T2 in the modMBSR arm. Surgical skill performance improved more at T2 in the modMBSR arm. **Burnout at 2 and T3 showed similar increase in both groups.** Cognitive control decreased more in the control arm at T2 and at T3 | Small sample size, use of volunteers, and execution at a single institution with its own unique issues and resources | National Institutes of Health and grantsfrom Larry L. Hillblom Foundation | None |  |
| Ireland et al (2017) | A randomized controlled trial of mindfulness to reduce stress and burnoutamong intern medical practitioners | Medical Teacher | To examine the effectiveness of a mindful-ness training intervention in reducing stress and burnout among intern doctors | Australia | Pre post study | Not stated | Not specified | Australia | Brisbane | Intern doctors | Intern/Foundation | None stated | 44 | Intern doctors completing an emergency department rotation in a major Australian hospital | 22-48 years (M+/-6.88, SD+/-4.79) | 10 sessions a mix mindfulness education and practice. Material was adapted from well-validated psycho-logical treatment programs (Mindfulness-Based StressReduction, Mindfulness-Based Cognitive Therapy, and Acceptance and Commitment Therapy | 10 weeks | Mindfulness | Participants undergoing the 10-week mindfulness training program reported greater improvements in stress andburnout relative to participants in the control condition. Significant reduction in stress and burnout was observed for partici-pants in the mindfulness condition. No such reductions were observed for participants in the control condition. | Small sample size, could not minimize any transfer of information on mindful-ness between conditions and thus, there is a possibilitythat the control participants benefited indirectly from themindfulness program |  |  |  |
| Fraiman et al (2022) | Effect of a Novel Mindfulness Curriculum on Burnout During Pediatric Internship: A Cluster Randomized Clinical Trial | JAMA Pediatrics | To examine whether a novel mindfulness curriculum implemented in the first 6months of internship reduces burnout | USA | Randomized control trial | 2017-2019 | 20 months | USA | Massachusetts | Paediatric interns | Paediatrics | 146 | 340 | Pediatric and medicine-pediatric in-terns training in study programs | 51 people were Age ≥30 y (15% of participants) | 7 hour-long sessions of a monthly mindfulness curriculum (Mindfulness Intervention for New Interns) and a monthly mindfulness refresher implemented during internship. The active control arm included monthly 1-hour sociallunches | 6 months, for a total of 7 sessions | Mindfulness | Both arms’ Maslach Burnout Inventory Human Services Survey emotional exhaustion scores were higher at 6 and 15 months than at baseline, but emotional exhaustion did not significantly differ by arm in multivariable analyses. A novel mindfulness curriculum did not significantly affect emotional exhaustion, burnout, empathy (Interpersonal ReactivityIndex’s Perspective Taking and Empathic Concern), or mindfulness (9-item Five Facet Mind-fulness Questionnaire) immediately or 9 months after curriculumimplementation. | Curriculum was not facilitated by an experienced mindfulness practitioner (curriculum relied on a scripted curriculumthat did not require a content expert to facilitate education. Intervention programs received the MINdI facili-tator manual and all necessary curriculum implementationsupplies.) |  |  |  |
| Martins et al (2011) | Impact of a brief interventionon the burnout levels of pediatric residents | Jornal de Pediatria | To estimate burnout prevalence among pediatric residents and to evaluate the impact of a brief intervention aimed at controlling burnout | Argentina | Randomized control trial | Not stated | Not stated | Argentina | Buenos Aires | Paediatric residents | Paediatrics | None stated | Experimental group (n = 37), control group (n = 37) | Medical residents in pediatrics from a tertiary care pediatric hospital. Medical students excluded | 27.3±1.4 years | The experimental group received a brief intervention consisting of two 2.5-hour workshops directed by mental health professionals, which covered repercussions of burnout syndrome on professional activity, recognition of risk indicators for burnout syndrome, and tools to cope (identification of strengths, coping behaviors, preventive and self-care behaviors) | 1 month | Workshop and group discussion | significant improvement was observed only in the experimental group, in the scores corresponding to depersonalization (p = 0.031). No effect on Emotional exhaustion or Personal accomplishment | 81% were female. Not clarified how many workshops were conducted within 1 month. Authors do not discuss limitations of own work. Does not mention whether participants and researchers were blinded. | Not stated | None |  |
| Kashat et al (2020) | Mindfulness Education for Otolaryngology Residents: A Pilot Study | OTO Open | to (1) implement a mindfulness-based wellness curriculum for otolaryngology residents, (2)determine the impact of a mindfulness-based curriculum onresident mood, and (3) examine the use of mindfulnessamong otolaryngology resident | USA | Pre post study | Not stated | Not stated | USA | Connecticut | Otolaryngology residents | Surgery | None stated | 8 | Otolaryngology residents | age rangeof 28 to 32 years | 6 week mindfulness course with weekly group meditations guided exclusivelyby the mobile Headspace application as part of a comprehensive wellness curriculum. Each session was approximately 10 minutes and covered 1 to 2 introductory meditations | 6 weeks | Digital or mobile health (mHealth) intervention, mindfulness | Changes in mood before and after each ses-sion were assessed using the validated Positive and Negative Affect Schedule (PNAS). Residents reported a statisticallysignificant decrease in postsession negative affect scores(P\.001). A moderate positive correlation was notedbetween mindfulness scores and presession positive mood(Pearsonr= 0.597,P\.001). This pilot study supports thefeasibility and impact of including mindfulness training aspart of a resident wellness curriculum | Pilot study with only 8 participants, lack of control group, and short duration | None | None |  |
| Ospina-Kammerer et al (2003) | An evaluation of the Respiratory One Method (ROM) in reducing emotional exhaustion among family physician residents. | International Journal of emergency mental health | To evaluate a particular burnout risk reduction method among family physicians within a family practice residency program | USA | Pre post study | Not stated | Not stated | USA | Florida | Family practice residents | Family Medicine (GP) | No dropouts | 24 (experimental n=14, control n=10) | Family practice residents | Range 27-46, mean 31, SD 5.98 | Respiratory One Method (ROM) which is a form of meditation that relies on verbalising the word one during exhalation. Designed to mitigate the impact of emotional arousal. Once per week for four consecutive weeks | 4 weeks | Meditation | The experimental group had a statistically significant lower emotional exhaustion score on Maslach Burnout Inventory | The authors do not detail the control intervention. | Not stated | Not stated |  |
| Lebares et al (2021) | Enhanced Stress Resilience Training in Surgeons: Iterative Adaptation and Biopsychosocial Effects in 2 Small Randomized Trials | Annals of Surgery | To determine the effects of ESRT (an iteratively adapted and tailored MBI) on perceived stress, executive cognitive function, psychosocial well-being (ie, burnout, mindfulness) | USA | Randomized control trial | 2016 and 2017 | 8 weeks | USA | San Francisco | mixed specialty PGY-1 volunteers | Mixed | 6 (due to dropout, conflicting timetable) | Intervention 46, control 43 | PGY-1 residents | Not stated | Enhanced stress resilience training (ESRT) during protected time. Training on moment-to-moment awareness of thoughts, emotions, and sensations, emotional regulation, conscious awareness of these cognitive control processes. 8 weekly 2-hour classes, focused on the development of mindfulness meditation skills using culturally acceptable language. In second RCT, similar however more explicitly applied to surgery, hospital-based work, and challenges of maintaining well-being during demanding training.  Control were of equal length and duration as the intervention, held during protected time, and involved group reading and discussion of lay articles pertaining to stressful and challenging aspects of surgical/medical training. | 8 weeks (in first control trial), 6 weeks in second. | Mindfulness | This pilot work suggests ESRT can variably benefit executive function, burnout, and physiologic distress in PGY-1 trainees, with potential for tailoring to optimize effects. Neither version of ESRT appeared to affect perceived stress. Higher executive function and mindfulness scores were seen in ESRT-1, and lower emotional exhaustion and depersonalization scores in ESRT-2, at pre-/post-intervention and/or 50-week follow-up (ESRT-1) or at 32-week follow-up (ESRT-2), compared to controls. | Small sample size. Generalizability of our findings is limited by the use of a resident population that was homogeneous in terms of training level (all PGY-1s), execution at a single institution, and the use of the same instructor for both trials. | Not stated | Not stated |  |
| Zazulak et al (2017) | The art of medicine: arts-based trainingin observation and mindfulness for fostering the empathic response in medical residents | Medical Humanities | To explore the impact of a course in arts-based visualliteracy and mindfulness practice (Art of Seeing) on the empathic response of medical residents engaged inobstetrics and gynaecology and family medicine training | Canada | Cohort study | October 2013and February 2014 | 3 months | Canada | Ontario | medical residents engaged in obstetrics and gynaecology and family medicine training | O&G, Family Medicine (GP) | None stated | 15 residents as intervention. 20 control | Medical residents engaged in obstetrics and gynaecology and family medicine training | mean age=30.56 years intervention, mean age=27.65 years control | Intervention group participated in thefour 3-hourArt of Seeingsessions at the MMA over the courseof 1 month. Multifaceted arts-based programme thatintegrates the facilitated viewing of art and dance, art-making, and mindfulness-based practices into apractitioner-patient context | 1 month | Mindfulness | The results indicated that programme participants improved in the Mindfulness Scale domains related to **self-confidence and communication, non-judgment of inner experiences, describe/self-expression,** relative to a group of control participants following the arts-based programme. Importantly, thematic qualitativeanalysis of the interview data revealed that theprogramme had a positive impact on the participants’perceived empathy towards colleagues and patients andon the perception of personal and professional well-being. | Generalisability - unclear if training curriculum will take up this art program, even if there were significant findingsacross the psychometric measurements. Small sample size. Does not mention control and intervention groups are similar in baseline characteristics. Small proportion of male participants. | ssociated MedicalServices (AMS) Phoenix Project Call to Caring Grant | None |  |
| Verweij et al., 2017 | Mindfulness-Based Stress Reduction for Residents: A Randomized Controlled Trial | Journal of General Internal Medicine | To evaluate the efficacy of Mindfulness-Based Stress Reduction (MBSR) in reducing burnout among medical residents. | Netherlands | Randomized control trial | 2017 | Not specified | Netherlands | Nijmegen | Medical residents | Medicine | 10 | 148 | All medical,surgical and primary care disciplines of the Radboud Univer-sity Medical Center Nijmegen. Excluded residents who had participated in an MBSR course previously | 31.2 (SD 4.6) | Mindfulness-Based Stress Reduction (MBSR) program. . Group-based MBSR courses, with sessions offered about three evenings a week. Participants prac-ticed formal mindfulness exercises including the body scan,yoga, and sitting and walking meditation. They receivedpsycho-education about stress, and were instructed to practicedaily at home for 45 min. The MBSR courses were taught by 11 differ-ent trainers, all of whom met the requirements of the good-practice guidance for teaching mindfulness-based courses | Eight weekly 2.5-h sessions in the eveningand a 6-h silent day during the weekend | Mindfulness | Nosignificant difference in emotional exhaustion was foundbetween the two groups. However, the MBSR groupreported significantly greater improvements thanthecon-trol group in personal accomplishment (p= 0.028), worry (p= 0.036), mindfulness skills (p=0.010), self-compassion (p= 0.010) andperspective-taking (empathy) (p=0.025). Noeffects were found for the other measures. Exploratory moderation analysis showed that the intervention out-come was moderated by baseline severity of emotionalexhaustion; those with greater emotional exhaustion didseem to benefit | The study was performed in a singlemedical university hospital in the Netherlands, which may limit generalizability. Participants were also self-selected, so the results may have been influenced byselection bias. Men and residents from the surgical spe-cialties were relatively underrepresented. The results are also limited to immediate post-intervention outcome. | Department for Evaluation,Quality and Development of Education of the Radboudumc | Dr. Speckens is clinical director of the RadboudUniversity Medical Center–Centre for Mindfulnes |  |
| Lases et al (2016) | Evaluating Mind Fitness Training and Its Potential Effectson Surgical Residents’ Well-Being: A Mixed Methods Pilot Study | World Journal of Surgery | To evaluate the impact of a mindfulness-based training program on the well-being of surgical residents | Netherlands | Cohort study | September 2012 to February 2014 | 14 months | Netherlands | Amsterdam | Surgical residents | Surgery | 2 residents from the intervention group, 21 from control group | 22 residents in the intervention group at baseline, 47 in the control group | Surgical residents in eight teachinghospitals, including one academic medical center | Not specified | Mind Fitness Training (MFT) program, grounded in the theory of mindful practice, which suggests that enhancing attention and self-awareness. Included various activities such as meditation, self-awareness exercises, and discussions. Delivered by a highly experienced trainer, who has a background in training top athletes and managers | 3-month training program consisting of five sessions | Mindfulness | The program aimed to yield improvements in residents' empathy, specialty satisfaction, stress perception, and focusing skills. Residents' feedback indicated that they found the training to be beneficial, particularly in terms of improving their ability to focus, manage stress, and become more aware of their mental state. In intervention-group, residents’ specialty satisfaction increased by 0.23 point on 5-point Likert scale (95 % CI 0.23–0.24,P<0.001) while stress scores decreased. | Small sample size Voluntary participation, which may lead to selection bias Difficulty in standardizing the training and measuring the effect of the trainer | Dutch Ministry of Health; the AcademicMedical Center, Amsterdam; and the Faculty of Health and LifeSciences of the University of Maastricht | Not stated |  |
| Chaukos et al (2018) | SMART-R: A Prospective Cohort Study of a Resilience Curriculum for Residents by Residents | Academic Psychiatry | To evaluate the feasibility and impact of the Stress Management and Resiliency Training Program for Residents (SMART-R) on burnout, fatigue, and depression in first-year residents in medicine and psychiatry. | USA | Pre post study | Not specified | 6 months | USA | California | first-year residents in medicine and psychiatry | Medicine, Psychiatry | 41% completed at least 10 of the 11 post questionnaires | 75 | PGY1 residents in medicine and psychiatry | Not specified | The Stress Management and Resiliency Training: Relaxation Response Resiliency Program curriculum, which included meditation, behavioral skills, and positive perspective-taking strategies across six hours of group-based instruction. Fcused on mind-body techniques, stress awareness skills (focusing on behavioral strategies for common thought distortions held by doc-tors), meaningful goal-setting techniques, and positiveperspective-taking approache | 6 hours, divided into three sessions over the first 6 months of the intern year | Workshop and group discussion | Negligible effect on burnout. There was no significantchange in perceived stress, worry, sense of personal accomplishment, coping skills,self-efficacy, mindfulness, or empathic perspective-taking. | Low survey response rate, lack of control group, and potential non-response, reporting, and selection bias. | PIRE Janssen Resident Award | Dr. Denninger receives support for unrelated investigator-initiated studies from Onyx Pharmaceuticals and materials from Basis Inc. |  |
| Saadat et al (2012) | Wellness program for anesthesiology residents: a randomized, controlled trial | Acta Anaesthesiologica Scandinavica | To evaluate the effects of a wellness program on anesthesiology residents' well-being | USA | Randomized control trial | 2006–2008 | 2 years | USA | California | First- to third-year anesthesiology residents | Anaesthetics | 2 | 58 | First- to third-year anesthesiology residents. residents who were randomized into the WIG and NTC-RT groups during the first year were not eligible to participate in the second year of the study | WIG: 30.7 (2.3), NTC-RT: 32.1 (4.8), NTC-RD: 31.1 (2.6) | Wellness intervention group (WIG), the no-treatmentcontrol group with release time (NTC-RT), and theno-treatment control group with routine duties(NTC-RD).   Wellness intervention was an evidenced-based intervention, based on Pearlin and Schooler’s hierarchy of coping mechanisms. Aimed at eliminating or modifying sources of stress so that continuing efforts to cope with a particular stressor are less needed, use of effective problem-solving and communication skills, minimizing the use of avoidance coping | 16 weeks - 16 1.5-h weekly sessions | Workshop and group discussion | Residents in the intervention group reported significantly fewer stressors in their role as parent, increased social support at work, greater problem-solving coping, and less anxiety compared with one or both control groups. Decreased use of avoidance coping and alcohol consumption were also noted but were not statistically significant. | Small sample size, lack of long-term follow-up assessments, reliance on self-reported measures, and no multimethod approaches to supplement self-report data | Foundation of Anesthesia Education and Research (FAER) | Not stated |  |
| Minichiello et al (2020) | Developing a Mindfulness Skills-Based Training Program for Resident Physicians. | Family Medicine | To assess the impact of a mindfulness training program on resident physician burnout and resilience | USA | Pre post study | 2016 | Not stated | USA | Wisconsin | family medicine residents | Family Medicine (GP) | Not stated | 17 | family medicine residents | Not stated | 10-hour mindfulness training over the course of 2 months, 5 sessions. Experienced Mindfulness-Based Stress Reduction teachers and a family physician/integrative health fellow cotaught the program. | 2 months | Mindfulness | Qualitative results identified multiple personal/professional benefits of participating in mindfulness training, and we found a statistically significant decrease in perceived stress and increase in mindful awareness from pre- to post intervention (P<.05). Surveys included the Maslach Burnout Inventory, Perceived Stress Scale Brief Resilience Scale (assessing one’s ability to bounce back from stress), and Five Facet Mindfulness Questionnaire | Small-scale observational study with no control group. Attendance was not tracked to support resident privacy, so we could not assess any association be-tween attendance and the quantita-tive measures over time. Not stated who developed the mindfulness program or how valid the mindfulness program is | The University of Wis-consin-Madison Department of Family Medicine and Community Health. UW Integrative Health program’s George Washington Institute for Spirituality and Health grant. National Center for Complementary and Integrative Health | Not stated |  |
| Feld et al (2006) | An evaluation of a support group for junior doctors working in palliative medicine. | The American journal of hospice & palliative care | To evaluate the effect of a Support Group for Junior Doctors Working in Palliative Medicine | UK | Cross-sectional study | Not stated | Not stated | UK | Hertfordshire | Palliative care junior doctors | Medicine | Not stated | 25 | Palliative care junior doctors in an inner-city hospice | 31 years, age range was 27-59 | N/A | N/A | Mentorship | 88% doctors found that a 1-hour session length was satisfactory. Most respondents (from 60% to 100% for the different specialties) thought that support groups would be helpful for all specialties listed apart from accident and emergency (8%). Ways in Which the Group Was Perceived as Helpful: Sharing experiences, Establishing relationships. Doctors commented on the importance of confidentiality. Some barriers - time constraints, unhelpful aspects of “the group dynamic,” | Small study sample, not detailed how these were selected | Boehringer-Ingelheim | Not stated |  |
| Weight et al (2013) | Physical Activity, Quality of Life, and BurnoutAmong Physician Trainees: The Effect of aTeam-Based, Incentivized Exercise Program | Mayo Clinic | To prospectively study the effects of an incentivized exercise program on physical activity (PA),quality of life (QOL), and burnout among residents and fellows (RFs) in a large academic medical center | USA | Cohort study | 2011 | 3 months | USA | Minnesota | Residents and fellows at Mayo | Mixed | 96 did not complete the exit survey | Total(N=628), Participants(n=245), Nonparticipants(n=383) | Residents and fellows at Mayo | 31.3 (SD 4.1) | Team-based, 12-week, self-directed incentivized exercise program. Points wereawarded on a weekly basis for self-reported exer-cise and gym attendance, survey completion, wellness examination completion, and improve-ments in objectivefitness parameters, such assubmaximal oxygen consumption, leg pressstrength, and body fat percentage | 3 months | Yoga and exercise | At the study conclusion, exercise program participants rated their QOL higher than non-participants (median, 75 vs 68;P<.001). Burnout was lower in participants than in nonparticipants, although the difference was not statistically significant (24% vs 29%;P=.17). A team-based, incentivized exercise program engaged 23% of RFs at our institution. After the program, participants had higher PA and QOL than nonparticipants who had equal exercise facility access. Residents and fellows may be much more sedentary than previously reported. | only23% of RFs in this cohort chose to participate in the intervention --> self selecting participants (participation biasand the Hawthorne effec) , more self driven, confounding factors? only 48% of exercise program participants met the Department of Health and Human Services Physical activity guidelines (an adult shouldparticipate inatleast150 minutes of moderate-intensity aerobic activity or 75 minutes of vigorous-intensity activity per weel) | Mayo Schoolof Graduate Medical Education | Not stated |  |
| Romcevich et al (2018) | Mind-Body Skills Training for Resident Wellness: A Pilot Study of a Brief Mindfulness Intervention | Journal of Medical Education and Curricular Development | To test the feasibility of a brief mind-body skills training (MBST) intervention, using in-person peer-led training supported by online modules, to decrease stress and burnout in pediatric resident physicians | USA | Pre post study | Not stated | Not stated | USA | Ohio | Residents at Nationwide Children’s Hospital in Ohio | Medicine, Paediatrics | None stated | 10 | Residents were categorical pediatrics and internal medicine-pediatric residents in their second through fourth years of postgraduate training. No specific selection criteria were required, only avail-ability and willingness to participate were required | 26-37 (mean age 29) | 4 weekly group mind-body skills training (MBST). basic skills sessions lasting 90minutes each, led by a resident with 5years of informal meditation and mindful movement experience. Sessions focused on open discussion of module content, sharing of participants’ mindfulness learning experiences between ses-sions, and hands-on teaching of MBST techniques, with most of the session time devoted to practice of the skills.  For the following 6months, the residents were offered optional monthly “main-tenance” group sessions, with the opportunity to join remotely via online group video chat | 6 months | Mindfulness | This pilot study demonstrates that a peer-led, short mixed-method mindfulness-based skills course may be a practical way to offer resilience and stress management training and improve wellness in busy pediatric residents.There was significant improvement in positive attitude, perceived stress, and resilience post intervention: Personal Achievement on the MBI (P=.002), perceived stress (P=.015), and resilience (P=.041) Follow-up evaluation also demon-strated significant improvement in burnout (depersonalization) and mindfulness. More than 75% of participants found the course worthwhile. | Most participants were women (70%). Small sample size. Because the intervention was completed in July, it may be relevant to note that the junior residents had completed their intern year immediately prior to the intervention. Their initial scores may reflect the stress accumulated over their intern year, or, alternatively, they may have felt a sense of renewal and accomplishment by having recently completed their internship | Nationwide Children’s Hospital Pediatrics Residency program | None |  |
| Nomura et al (2016) | Limitation of duty hour regulations for pediatricresident wellness: A mixed methods study in Japan | Medicine | To evaluate the mental health of pediatric residents following the implementation of the overnight call shift (OCS) system - residents get to take8hours off work after the morning round, rather than being on-call after their usual dayshift, resulting in some of them working for more than 30 consecutive hours. | Japan | Cross-sectional study | 2013 | Not specified | Japan | Aomori | Paediatric residents | Paediatrics | None stated | 41 | Inclusion criteria included pediatric residents at National Center for Child Health and Development. Exclusion criteria were not explicitly mentioned. | Mean 29 years | Implementation of an overnight call shift (OCS) system | N/A | Duty hour restriction | There was no statistical difference in age, resident year,postgraduate year, working hours, frequency of overnight call,and frequency of off-duty days between residents with depressivesymptoms and those without depressive symptoms. orkingconditions, such as frequency of overnight calls, frequency ofoff-duty days, and working hours, were not statisticallyassociated with burnout. | Small sample size, single-institute nature, and limited generalizability | Not stated | Not stated |  |
| Foster et al (2012) | RAFT (Resident Assessment Facilitation Team): Supporting Resident Well-Being Through an Integrated Advising and Assessment Process | Family Medicine | To investigate the impact of the Resident Assessment Facilitation Team (RAFT) process on resident well-being as part of the Preparing the Personal Physician for Practice (P4) innovation | USA | Cohort study | 2007 to 2010 | Not specified | USA | Pennsylvania | Residents in the 2007 to 2010 PGY-1 cohorts | Mixed | None stated | Not specified | Residents who entered as PGY-1 residents in 2007 and graduated in 2010, and residents in the 2008 to 2010 PGY-1 cohorts | Not specified | Implementation of the RAFT process. The Resident Assessment Facilitation Team (RAFT) is an innovative feedback process that was implemented as part of the Preparing the Personal Physician for Practice. Itinvolves small-group dialogue and replaces the traditional semi-annual, faculty-wide review of resident performance. It assumes residents’ capacity for self-direction, involvement in educational planning, and a decreased need for oversight as they mature. It is designed to model a dialogic, respectful, and inclusive conversation, reinforcing values of relationship-centeredness that are core features of the residency curriculum and culture | Not explicitly mentioned | Improving resources | The Arizona Integrative Outcome Scale (AIOS) is a visual analogue scale used to assess the global sense of well-being. The study did not find significant differences in AIOS scores between the pre- and post-RAFT cohort groups | Small population, voluntary sampling procedures, inability to establish a direct causal relationship between the RAFT process and outcome measures of resident well-being | The Dorothy Rider Pool Health Care Trust | Not stated |  |
| Babbar et al (2019) | Addressing Obstetrics and Gynecology Trainee Burnout Using a Yoga-Based Wellness Initiative During Dedicated Education Time | Obstetrics & Gynecology | To assess the feasibility and efficacy of implementing a yoga-based wellness program during trainee education time and its influence on trainee burnout, depression, anxiety, stress, mindfulness, and perceived utility. | USA | Pre post study | Conducted between late October and mid-December 2017 | 8 weeks | Saint Louis University, St. Louis, MO | Midwest | Obstetrics and gynecology residents and five maternal–fetal medicine fellows | O&G | None stated | 24 | Voluntary participation, open to obstetrics and gynecology residents and maternal–fetal medicine fellows | Mean age of 29.6 years | 8-week wellness program consisting of weekly yoga classes, nutrition, and physical challenges | 8 weeks | Yoga and exercise | Baseline demographics showed that the majority of participants were sedentary or infrequently engaged in exercise before the program. At baseline, participants exhibited moderate emotional exhaustion, low to moderate depersonalization, and a high sense of personal accomplishment, collectively indicating a low degree of burnout. After the program, there was a significant reduction in depersonalization and anxiety, as well as in systolic and diastolic blood pressure. The study also revealed positive feedback from participants, indicating increased camaraderie, appreciation, motivation, and overall training experience. | Low participation rate, potential bias due to the nature of rotations, duty hour limitations, and holiday seasons. Relatively low degree of burnout, anxiety, depression, and stress at baseline. | Not stated | None stated |  |
| Ripp et al. (2016) | A Randomized Controlled Trial to Decrease Job Burnout in First-Year Internal Medicine Residents Using a Facilitated Discussion Group Intervention | Journal of Graduate Medical Education | To determine if a facilitated discussion group intervention could reduce incident burnout among first-year internal medicine residents | USA | Randomized control trial | Conducted between June 2013 and May 2014 | 11 months | Icahn School of Medicine at Mount Sinai, New York | New York | First-year internal medicine residents | Medicine | 12 out of 51 residents did not complete both surveys | 51 | Incoming first-year internal medicine residents at the Icahn School of Medicine at Mount Sinai | Not specified | Twice-monthly theme-based discussion sessions led by psychotherapist facilitators for the intervention group. Psychotherapy was not part of theintervention. Control group received lunch vouchers. | 11 months | Workshop and group discussion | A facilitated discussion intervention modeled after asuccessful program used to decrease job burnout inpracticing physicians was not found to be effective inresident trainees.The intervention did not decrease burnout in resident physicians; more residents in the intervention group had high depersonalization scores at the study end. This is possibly due to the limitations of thedesign of the intervention in this population or theunique challenges of residency training comparedwith posttraining practice, particularly reduced au-tonomy and control of schedules and workload. | The intervention did not effectively free residents from clinical responsibilities; the study was underpowered due to the smaller than anticipated class size | Feldstein Medical Foundation | None |  |
| Prins et al (2007) | The role of social support in burnout among Dutch medical residents | Journal: Psychology, Health & Medicine | To examine the satisfaction of medical residents with social support and the correlation between social support and burnout. | Netherlands | Cross-sectional study | 2003 | Not specified | Netherlands | Groningen | Medical residents | Medicine | N/A | 158 | Medical residents in the University Medical Center Groningen | Not specified | Social support (emotional, appreciative and informative) received from supervisors, fellow medical residents, nurses and patients | N/A | Social support | Significantly more dissatisfaction with emotional, appreciative, and informative support received from supervisors compared to fellow residents, nurses, and patients. Dissatisfaction with emotional support from supervisors had a significant effect on emotional exhaustion, and dissatisfaction with appreciative support from supervisors had an effect on depersonalization. The best predictor of burnout appeared to be dissatisfaction with emotional support received from supervisors. Our results suggest that intervention programs should not only focus on the medical residents, but also on the supervisors to improve their supportive skills. | Small sub-samples, cross-sectional design | Not stated | Not stated |  |
| Forbes et al (2020) | Resilience on the Run – an evaluation of a wellbeing program for medical interns | Internal medicine journal | To evaluate the acceptability and effectiveness of a resilience and wellbeing program for medical interns | Australia | Cohort study | Not stated | Not specified | Australia | Queensland | Medical interns | Medicine | Not explicitly stated but implies large number of dropouts | 24 intervention, 29 control | All participants had graduated from an Australian medical school, medical interns | mean 26.99 years | four ninety-minute group sessions - Resilience on the Run (RoR) is an innovative, evidence-based program specifically designed for junior doctors developed by a psychiatrist - provides practical skills and strategies for integrating these into their working life, including mindfulness and meditation. | 360 min | Mindfulness | The comments from the participants regarding the Resilience on the Run (RoR) program were generally positive, with close to 90% agreeing that the sessions increased their awareness of positive strategies they can use to maintain their mental health and wellbeing. The participants found the ideas to be impactful and appreciated the concepts and ideas presented during the sessions. No statistical comparison due to insufficient participant completion of survey instruments. | small participant numbers and the even smaller number of paired responses | AMA Queensland Foundation | Not stated |  |
| Gopal et al. (2005) | Burnout and Internal Medicine Resident Work-Hour Restrictions | Archives of Internal Medicine | To evaluate rates of burnout in internal medicine residents before and after the implementation of new work-hour restrictions | USA | Pre post study | May 2003 to May 2004 | 1 year | USA | Colorado | Internal medicine residents | Medicine | Response rate of 87% in 2003 and 74% in 2004 | 139 residents in 2003, 143 residents in 2004 | Internal medicine residents | Majority in the 26-30 year age group | Implementation of work-hour restrictions, limiting residents to work less than an average of 80 hours per week and no more than 30 hours of continuous duty for patient care and educational obligations | N/A | Duty hour restriction | Decrease in high emotional exhaustion from 42% to 29% (P=.03), fewer residents with highdepersonalization (61% vs 55%;P= .13) and fewer resi-dents with a positive depression screen (51% vs 41%;P= .11), however decrease in attendance at educational conferences from 18.99 to 15.56 per month (P=.01), decrease in overall residency satisfaction from 76.3 mm to 70.3 mm on a 100-mm visual analogue score (P=.02) | Potential recall bias, inaccuracies of self-report, unknown effect of burnout on reporting, limitations in study power to detect small to moderate effects | General Internal Medicine Division of the Department of Medicine at the University of Colorado Health Science Center | None |  |
| Milstein et al., (2009) | Burnout assessment in house officers: Evaluation of an intervention to reduce stress | Medical Teacher | To measure burnout in house officers and establish whether utilization of a psychotherapeutic tool individually by physicians reduces symptoms characteristic of burnout | USA | Randomized control trial | First academic quarter of 2003 | 3 months | USA | Sacramento, CA | Paediatric house offices | Paediatrics | Not explicitly mentioned | 33 potential subjects; 15 enrolled with 8 randomized to a control group and 7 to a study group | Pediatric house officers from the UCD Pediatric Residency Training Program | Not specified | The study group received 45-minute instruction in the use of a psychotherapeutic technique, BATHE (Background, Affect, Trouble, Handling, and Empathy). Participants were encouraged to use the technique approximately three times per week over the subsequent three-month period | 3 months | Mindfulness | No significant differences in burnout symptoms between the control and intervention groups based on the Maslach Burnout Inventory (MBI) scores. Qualitative interviews revealed that subjects experience stress and already utilize some elements of the BATHE tool. | The study sample size was small, and the intervention may not have been used systematically by participants. | Not stated | None stated |  |
| Barrack et al., (2006) | Effect of Duty Hour Standards on Burnout among Orthopaedic Surgery Residents | Clinical Orthopaedics and Related Research | To quantify quality of life measures including burnout, general health, and relationship issues among orthopaedic surgery residents and faculty, and to assess the impact of Accreditation Council on Graduate Medical Education (ACGME) duty hour standards on these measures. | USA | Cohort study | The first survey was completed in 2002, and the second survey was administered in 2005. | Approximately 3 years between the first and second survey. | USA | Missouri, LA | Orthopaedic surgery residents | Surgery | Not explicitly mentioned | 55 residents | Orthopaedic surgery residents (and faculty members however this is excluded from analysis) | Not specified | Implementation of ACGME duty hour standards, which include an 80-hour workweek limit averaged over four weeks, at least one day out of seven free of clinical and educational activities, a continuous work hour limit of 24 hours plus an additional 6 hours of administrative time, and an on-call limit of every third night. | The duty hour standards were in effect for two years at the time of the second survey | Duty hour restriction | Resident duty hour limitation was associated with improvement in objective measures of burnout Improvement in resident scores for personal accomplishment (p < 0.01) Lower scores for emotional exhaustion among residents (p ≈ 0.056). Somewhat lower depersonalization scores among residents (p ≈ 0.14) | Not possible to determine what percentage of faculty and residents completed both surveys due to the voluntary and anonymous nature of the surveys. Small sample size, only two training programs involved, different participants in the pre- and post-intervention surveys, and potential other changes in training programs that could have impacted the scores | Orthopaedic Research and Education Foundation | None stated |  |
| Martini et al (2006) | Comparison of Burnout Among Medical Residents Before and After the Implementation of Work Hours Limits | Academic Psychiatry | To assess whether the implementation of work hour limits is associated with a lower prevalence of medical resident burnout. | USA | Cross-sectional study | Survey mailed between February and May 2004 | Not explicitly stated, but the survey distribution occurred over a period of approximately 3 months. | USA | Michigan | Medical residents and interns in general surgery, internal medicine, family medicine, obstetrics/gynecology, pediatrics, and psychiatry | Mixed | Not explicitly mentioned | 118 | All residents and interns were eligible except for the first author (a PGY-4 psychiatry resident at the time of conducting the study). | Not stated | Implementation of work hour limits by the ACGME in July 2003 | Not applicable as the intervention was a policy change implemented prior to the study | Duty hour restriction | First-year residents reported a 43% burnout prevalence, significantly lower than the 77% burnout prevalence among first-year residents prior to the implementation of work hour limitations. Burnout in this study was measured using the Maslach Burnout Inventory (MBI) | Low response rate (31%), potential response bias, and residents' probable knowledge of the study's purpose, which could influence their responses. | Not stated | None stated |  |
| Shea et al., 2014 | Impact of Protected Sleep Period for Internal Medicine Interns on Overnight Call on Depression, Burnout, and Empathy | Journal of Graduate Medical Education | To examine the impact of a protected sleep period on the emotional well-being of internal medicine interns, specifically looking at depression, burnout, and empathy. | USA | Randomized control trial | 2009–2010 | 1 year | USA | Phyladelphia | Internal medicine interns | Medicine | 3 | 103 | Interns and senior medical student subinterns serving on either an internal medicine rotation | Not specified | A 5-hour period of protected sleep from 12:30 AM to 5:30 AM during on-call nights. | The intervention was applied during the interns' 4-week rotation schedule. | Duty hour restriction | No significant differences in end-of-rotation assessments of burnout, depression, or empathy between the groups with and without protected sleep. A protected sleep period produced fewconsistent improvements in depression, burnout, orempathy, although depression was already low atbaseline. | Single institution study, potential insensitivity of measurement scales, not powered to detect changes in depression or burnout scores, no control for the rotation from which the intern had come, and interns are no longer allowed to work more than the hours specified in the study. | grant VA HSR&D EDU 08-429 (K.G.V.) | None |  |
| Antiel et al., 2013 | Effects of Duty Hour Restrictions on Core Competencies, Education, Quality of Life, and Burnout Among General Surgery Interns | JAMA Surgery | To assess surgical interns' views on the impact of new duty hour regulations on their training and patient care, examine changes in these views from the beginning to the end of their internship, and measure the current status of their quality of life and level of burnout near the end of their intern year | USA | Pre post study | Baseline data from the beginning of the intern year (2011) and follow-up data near the completion of the intern year (May 2012) | Approximately one year | USA | Northeast, Midwest, South, and West regions of the United States | Surgical interns | Surgery | 2 residents were no longer in their respective programs at the end of the year | 213 eligible surgical interns, with 156 completing the survey in 2012 (73% response rate) and 179 completing the survey in 2011 (83% response rate) | All surgical interns at the 11 selected general surgery residency programs were eligible to participate | Majority younger than 29 years | Implementation of the new ACGME duty hour restrictions | Not applicable as the study measures the impact of already implemented duty hour restrictions | Duty hour restriction | The first cohort of surgical interns to train under the new regulations report decreased continuity with patients, coordination of patient care, and time spent in the operating room. Furthermore, suboptimal quality of life, burnout, and thoughts of giving up surgery were common, even under the new paradigm of reduced workhours: High levels of emotional exhaustion and depersonalization reported weekly, indicating burnout, 1 in 7 interns considered leaving surgery weekly | Attitudinal associations may not be stable over time; the study only assessed postgraduate year 1 residents; surveys were blinded, precluding paired analyses of responses from 2011 to 2012 | Not stated | None |  |
| Goitein et al., 2005 | The Effects of Work-Hour Limitations on Resident Well-being, Patient Care, and Education in an Internal Medicine Residency Program | Archives of Internal Medicine | To investigate the effects of Accreditation Council for Graduate Medical Education (ACGME) work-hour limitations (WHLs) on resident well-being, patient care, and education. | USA | Pre post study | Surveys sent in February and March 2004 | Not specified | USA | Washington | Internal medicine residents | Medicine | Not explicitly mentioned, but the response rate was 73%. | 161 residents were surveyed, 118 responded | Not specified, but the study targeted internal medicine residents at a specific institution. | Not stated | Implementation of ACGME work-hour limitations | the study does not specify the exact duration of residents' exposure to WHLs before the survey. | Duty hour restriction | 84% of residents reported a positive effect on well-being due to work hour limitation (WHL)s. A decrease in emotional exhaustion from 53% to 40% (P = .05). Career satisfaction increased from 66% to 80% (P = .02). 37% reported a negative effect of WHLs on patient care, 29% positive, 34% neutral. 47% reported a negative effect on their education, 32% positive, 21% neutral. 65% of residents overall approved of WHLs. | The study may have been limited by survey bias, the self-reported nature of the data, and the inability to isolate the effects of WHLs from other factors. | Dr. Goitein received a National Research Service Award training grant from the Agency for Healthcare Research and Quality, Rockville, Md. | None |  |
| Winkel et al (2010) | No Time to Think: Making Room for Reflection inObstetrics and Gynecology Residency | Journal of Graduate Medical Education | To evaluate the feasibility of designing and implementing a reflective writing program within an obstetrics and gynecology curriculum and to create a model for other institutions to include programs for reflective activities in resident curricula. | USA | Cohort study | 2008-2009 | 1 year | USA | New York | Obstetrics and gynecology residents | O&G | One resident was not eligible due to maternity leave, and another had a prolonged absence due to illness. | 20 residents were scheduled to participate; 18 participated in the study. | ll residents in obstetrics and gynecology, from interns to fourth-year chief residents, were scheduled to participate. Exclusion criteria are not explicitly mentioned, but it can be inferred that residents on maternity leave or with prolonged illness were excluded. | Not stated | A series of six 1-hour-long reflective writing workshops.  To tailor thecourse content to relevant issues for the residents, anintroductory session included a structured focus group,during which the residents generated a list of topics they feltwere important to them that fell outside of their standardcurriculum - top 6 subjects were used as topics forthe writing workshops. Workshops included ashared reading of a short story, followed by discussion andthen a reflective writing exercise related to the topic. Designed by psychologist. | 6 weeks | Workshop and group discussion | Qualitative outcomes: Residents reported enjoying the workshops and feeling that they influenced their residency experience. However, many could not make the connection to their work with patients.Quantitative outcomes: Burnout and empathy scores, measured by the Maslach Burnout Inventory and the Interpersonal Reactivity Index, showed a nonsignificant trend toward preserved empathy and reduced burnout among participants. | Small size, unequal and nonrandomized participant groups, more junior residents participating than senior residents, and potential selection bias due to affinity for reflective work. | Not stated | None stated |  |
| Maher et al., 2013 | Stress training for the surgical resident | The American Journal of Surgery | To evaluate the efficacy of an educational program designed to improve surgical resident performance during stressful scenarios | USA | Cohort study | Between January 2011 and May 2011 | 5 months | USA | Philadelphia | 1st- and 3rd-year surgical residents | Surgery | Not stated | 26 (11 in the experimental group and 15 in the control group) | Exclusion criterion was participation in the study design | Not stated | Nine total hours of stress training were conducted during protected, predesignated educational conference time and had a 3-part focus: (1) the identification of individual stress triggers; (2) the identification of individual stress management techniques used before stress training sessions and instruction in 4 specific skills; and (3) the application of these techniques to surgical situations. | intervention took place over 3 consecutive Wednesdays with 3 hours per session | Workshop and group discussion | Both groups then completed a simulation during which stress was evaluated using objective and subjective measures, and resident performance was graded using a standardized checklist There was a trend toward improved performance scoring but no difference in anxiety levels after stress training. However, 91% of residents rated the stress training as valuable. Performance checklist scores were 5% higher in the experimental group than the control group (P = .54). No change in anxiety state according to the State Trait Anxiety Inventory (P = .34) or in heart rate under stress (P = .17) between groups | Allocation bias due to non-randomization Study underpowered, requiring more participants for robust data Flaws in the design of both the intervention and the testing Measurements used to evaluate stress are imperfect (State Trait Anxiety Inventory, heart rate). The study acknowledged that the development of a global stress management skill set requires more time than the 9 hours provided in the program. | Surgical Education via CESERT grant (number 10-01) | None |  |
| Runyan et al., 2016 | Impact of a family medicine resident wellness curriculum: a feasibility study | Medical Education Online | To evaluate the impact of a new, 1-month wellness curriculum on burnout, empathy, stress, and self-compassion among second-year family medicine residents. | USA | Pre post study | Not specified | 1-month intervention with a 3-month follow-up period | USA | Massachusetts | Second-year family medicine residents. | Family Medicine (GP) | None | 12 | Not explicitly stated in the provided context. | Not stated | A behavioral science facultymember (CR) implemented the wellness curriculum emphasizing competencies around leadership, focusing on teaching skills to cultivate mindfulness and self-compassion to enhance empathy and reduce stress. | 2h every week for 4 weeks | Mindfulness | This feasibility project demonstrates that a wellness curriculum can be executedwithin a residency with promising results and modest butdedicated faculty support. Positive trends on mean scores of all measures, particularly the Mindfulness Scale of the Self-Compassion Inventory and the Jefferson Empathy Scale. The small sample size resultedin lack of sufficient power to detect statistically significantdifferences pre/post-intervention. | Small sample size and lack of sufficient power to detect statistically significant differences pre/post-intervention. | The authors report no external funding source for this study. | The authors report no conflict of interest. |  |
| Brennan et al., 2015 | Designing and implementing a resiliency program for family medicine residents | The International Journal of Psychiatry in Medicine | o design and implement a resiliency program aimed at combating burnout and promoting wellness and resilience among family medicine residents. | USA | Cohort study | The article was published in 2015; the study year is not explicitly stated. | The study duration is not explicitly stated, but data was collected quarterly and at the end of the academic year | USA | Ohio | Family medicine residents, with a majority being international medical school graduates | Family Medicine (GP) | None stated | Intervention group with 10 residents, control group with 13 residents | Not specified. | Not specified | A resiliency program including interactive, experiential sessions on personal and professional values, self-awareness, stress management, health behavior improvement, time management, and fostering supportive relationships. System changes such as mindfulness meditation, healthier food options, and exercise equipment were also implemented. | The intervention seems to be conducted over the course of an academic year, with ongoing sessions and system changes. | Mindfulness | Initial scores on the Maslach Burnout Inventory revealed that 22% of residents rated high in emotional exhaustion, 9% in depersonalization, and 48% low in personal accomplishment. The effectiveness of the program is being evaluated through standardized psychological inventories. Results to date show excellent acceptance of the program by trainees, increased consumption of nutritious foods, more personal exercise, and self-reported decreased overreactions to stress. | Major challenges include scheduling difficulties for residents to attend sessions due to rotations, vacations, and sickness. The study's limitations are not fully detailed in the provided context. | Academy of Educators Grant at the University of Toledo Medical Center | None declared |  |
| Wen et al (2017) | Encouraging Mindfulness in Medical House Staff via Smartphone App: A Pilot Study | Academic Psychiatry | To assess how a self-guided, smartphone-based mindfulness app (Headspace) affects wellness as measured by prevalidated surveys among resident physicians. | USA | Pre post study | April 2015 to August 2016 | 16 months | USA | California | Residents in the departments of general surgery, anesthesia, and obstetrics and gynecology | Surgery, anaesthetics, O&G | 13 out of 43 participants did not complete the study | 43 enrolled; 30 completed two or more surveys and were included in the analysis | Male and female residents from Stanford University Hospital and Clinics, age > 21 years; no exclusion criteria mentioned | > 21 years | Use of the Headspace app, a self-guided, smartphone-based mindfulness app | 30 days | Digital or mobile health (mHealth) intervention, mindfulness | Surveys measuring mood (PANAS) and mindfulness (FMI) were taken at baseline, 2 weeks, and 4 weeks. The study found a significant increase in mindfulness and a positive trend in positive affect, but no change in negative affect. Mixed-effect multivariate modeling showed positive changes in both the Positive Affect Scale and the Freiburg Mindfulness Inventory scores with increasing app usage, while the Negative Affect Scale remained unchanged. | Self-guided app usage, homogenous study subject population, insufficient subjects to perform stratified analysis by specialty, lack of control group, potential influence from the Hawthorne effect | None | The authors declare no conflict of interest |  |
| Mache et al., 2017 | A pilot study evaluation of psychosocial competency training for junior physicians working in oncology and hematology | Psycho‐Oncology | To evaluate the outcomes of psychosocial competency training for junior physicians working in oncology and hematology medicine | Germany | Randomized control trial | Not specified | 36 weeks | Germany | Hamburg | Junior physicians working in oncology and hematology with an average working experience of 1.2 years | Medicine | Total dropout rate from randomization to analyses was 10%. Specific reasons included illness and participants not showing up. | 80 German-speaking employed junior physicians | Regular access to the Internet, Working full time in the hospital Working experience of max. two years, Being able and willing to participate for the next 36 weeks, Agreement to complete the, questionnaires, No prior knowledge of or experience with a mental health promotion training | Mean age 28.1 years (Intervention Group), 27.6 years (Waitlist Control Group) | Psychosocial competency training combined with cognitive behavioral and solution-focused counseling. Training content included work‐ related self‐care strategies, problem‐solving techniques solution‐focused counselling. The outcomes studied were changes in work‐related stress, emotional exhaustion, emotion regulation, and job satisfaction | 12 weekly sessions of 1.5 hours each | Workshop and group discussion, counseling | Intervention group reached a decrease in perceived job stress and emotional exhaustion. Self‐perceived improvements were also obvious regarding enhanced emotion regulation skills. Future oncologists valued the intervention with high scores for training design, content, received outcome, and overall training satisfaction. | Small sample size, limited to a pilot study, follow-up measurements only included 3 follow-ups in a period of 36 weeks, reliance on self-report measures, potential for positive bias within the study group | Not stated | no conflict of interest. |  |
| Bentley et al., 2018 | Relational Mindfulness for Psychiatry Residents: a Pilot Course in Empathy Development and Burnout Prevention | Academic Psychiatry | To pilot an empathy training course based in relational mindfulness and assess the impact on burnout and empathy among first-year psychiatry residents. | USA | Pre post study | Not specified | 8 weeks | USA | Southeast | First-year psychiatry residents (PGY-1) | Psychiatry | None stated | 7 | All seven PGY-1 residents at the institution were included; no specific exclusion criteria mentioned | Not specified | An eight-week course integrating relational mindfulness and empathy training, consisting of 1.5-hour sessions each week. The course was adapted from the literature on mindfulnesstraining in healthcare and from the course directors’trainingsin mindfulness-based stress reduction and motivationalinterviewing. | 8 weeks | Mindfulness | Significant improvement in empathy (p = .02); downward trend in all three burnout subscales (emotional exhaustion, depersonalization, personal accomplishment), though not statistically significant. Overall, the PGY-1s reported an increased awareness of their cognitive and emotionalexperiences and stated that the skills learned in the program increased their ability to care for themselves, their patients, and theirfamilies. | Small sample size, lack of a control group, reliance on self-report measures, potential social desirability bias. | Not stated | None |  |
| Mache et al., 2016 | Mental health promotion for junior physicians working inemergency medicine: evaluation of a pilot study | European Journal of Emergency Medicine | To implement and evaluate a mental health promotion program for junior physicians working in emergency medicine. | Germany | Randomized control trial | 2016 | 6 months of follow-up | Germany | Hamburg | Junior physicians working in emergency medicine with less than 3 years of experience | Emergency medicine | Of the 70 physicians enrolled, 72% of the intervention group completed all 12 training sessions. Attrition rates were 2% at survey 2, 5% at survey 3, and 11% at survey 4 for the intervention group | 70 junior physicians | Participation inclusion criteria: Employment in emergency medicine, full-time work in the hospital, less than 3 years of working experience, ability and willingness to participate, agreement to complete questionnaires, email access, and internet access.  Exclusion criteria: Psychiatric illness, taking psychiatric drugs, engaging any counseling service, and parallel use of psychosocial counseling. | Mean 27 years (SD = 2.4) | A mental health promotion program involving problem-focused and emotion-focused coping skills, cognitive behavioral, and solution-focused counseling in team training. Two qualified psy-chologists, both trained in cognitive behavioral as well assolution-focused work, performed the training. The training sessionsincluded psychoeducation (theoretical input, watchingvideos, oral group discussions, experiential exercises, andhome assignments). | 90-minute sessions over 3 months | Workshop and group discussion, counseling | Significant reductions in perceived stress and emotional exhaustion in the intervention group compared to the control group at all follow-up points. Medium between-group effect sizes for perceived stress were observed at T1 (Cohen’s d = 0.69), T2 (Cohen’s d = 0.61), and T3 (Cohen’s d = 0.57). Secondary outcomes also showed significant between-group effects for emotional exhaustion and emotion regulation competencies. NB - outcome measures included the Perceived StressQuestionnaire (PSQ), the Copenhagen PsychosocialQuestionnaire (COPSOQ), the Maslach Burnout Inventory,the Utrecht Work Engagement Scale (UWES), and theEmotion Regulation Skills Questionnaire-27 (ERSQ-27) | Small sample size, lack of formal sample size calculations, limited external validity, 6-month follow-up limiting long-term effect measurement, and reliance on self-report measures | Not stated | no conflict of interest. |  |
| Bragard et al., 2009 | Efficacy of a communication and stress management training on residents’ self-efficacy, stress to communicate, and burnout: A randomised controlled study | Journal of Cancer Education | To evaluate the impact of a communication and stress management training program on medical residents' self-efficacy, stress levels when communicating in clinical settings, and burnout. | Belgium | Randomized control trial | Not specified | 10 months | Belgium | Not specified | Medical residents working in the oncology field. | Medicine | 11 medical residents were excluded from the analyses because they did not complete the assessment procedure after training, and 6 were excluded for attending less than one hour of the training. | 96 | Medical residents had to speak French, show an interest in psychological training, and be willing to participate in the training program and its assessment procedure. | intervention mean 28.3 years (SD=3.0). Control group 28.1 years (SD=2.2). | A 40-hour training program focused on communication skills in cancer care, including a 30-hour communication skills component and a 10-hour stress management component. The stress management skills trainingfocused on four topics: detection of job stressors and stress outcomes, relaxation techniques, cognitive restructuring and time management. | 40 hours | Workshop and group discussion | Results showed a statistically significant increase in self-efficacy and decrease in stress to communicate. No changes were noted in burnout | Statistically significant differences were found at baseline between the intervention and control groupconcerning specialtyand previous communication skills training. Unclear whether the stress management classes are validated by psychologists. | The "Fonds National de la Recherche Scientifique - Section Télévie" of Belgium and the C.A.M. training and research group | No competing interests declared |  |
| Taylor et al., 2016 | A Mindfulness Intervention for Residents: Relevance for Pediatricians | Pediatric Annals | To examine the feasibility and impact of a brief mindfulness intervention using a free smartphone application with a resident population. | USA | Pre post study | Not specified | Not specified | USA | Chicago | Pediatric residency program participants | Paediatrics | Of the 33 enrolled participants, 31 completed the initial survey and 11 completed the follow-up survey. | 33 | Pediatric residency program participants | Not specified | Use of the free smartphone application Headspace to complete a 10-day program in mindfulness meditation | 10min x 10 sessions over 10 days | digital or mobile health (mHealth) intervention, mindfulness | Increased percentage of residents perceived mindfulness as a useful intervention for patients; statistically significant increase in the number of residents who planned to discuss mindfulness as a therapeutic option for their patients (61% from 48%, t = 2.7078). No statistically significant change in abbreviated Maslach Burnout Inventory (aMBI) scores from pre- to post-study groups | Small sample size, possible selection bias, reliance on self-reported data, majority of the mindfulness sessions completed on participants' own time. | Oberweiler Foundation | No competing interests declared |  |
| Ghetti et al., 2009 | Burnout, Psychological Skills, andEmpathy: Balint Training in Obstetricsand Gynecology Residents | Journal of Graduate Medical Education | To assess burnout, behavioral-medicine skills, and empathy among obstetrics and gynecology residents before and after the implementation of Balint trainin | USA | Pre post study | 2005 | 12 months | USA | Pittsburgh | Obstetrics and gynecology residents | O&G | None | 17 | Obstetrics and gynecology residents | Mean age 28 ± 2.2 years | Mandatory Balint group training as part of the residency curriculum - a way to discuss cases or situations arising in practice, which have aroused feelings in the trainees | two 1-hour group sessions once a month duringprotected, mandatory educational time over 12 months | Debrief | Burnout: 70% of residents reported high burnout scores at baseline, unchanged at 12 months. Empathy: Jefferson Scale of Physician Empathy scores remained unchanged at 12 months The residents demonstrated improved confidence and ability in handling the psychological aspects of patient care after 12 months of Balint training: with significant improvement in 3 individual Psychological Medicine Inventory questions --> i.e.improvement in ability to use consultation, ability to makeappropriate treatment decisions based on patients’ psychological needs, and ability to be psychologicallytherapeutic with patients | Small sample size, potential selection bias, lack of control group, reliance on self-report measures, and short duration to observe changes in complex characteristics like empathy. Unclear whether Psychological Medicine Inventory is a validated measure. |  |  |  |
| McCue et al (1991) | A Stress Management Workshop Improves Residents' Coping Skills | Archives of Internal Medicine | To assess the effectiveness of a stress management workshop in improving coping skills among medical residents | USA | Cohort study | 1991 | Not explicitly stated, however the study measured outcomes 8 weeks apart | USA | Massachusetts | Internal medicine and pediatrics residents | Medicine, Paediatrics | Not explicitly mentioned in the given context | 43 volunteered for the workshop, 21 in the nonintervention group | Residents who could be released from their usual work to attend the workshop; control subjects who could not be freed of clinical responsibilities | 36 participants in 26-29y range | A stress management workshop designed specifically for medical residents. The primary aim of the workshop was to teach participants effective strategies for managing the stresses associated with medical practice: Personal Management Skills, Relationship Skills, outlook skills, Stamina Skills. | 4h | Workshop and group discussion | All 21 individual ESSI Stress Systems Instrument scale items showed improvement for the workshop participants (vs only 8 in control). There was statistical improvement in Ability to cope with pressure and self-esteem. The workshop group also showed improvement in the MBI emotional exhaustion subscale. | on-randomized control group, potential "workshop high" effect, self-administered tests could increase awareness of stress independently, and the nonintervention group was not randomly selected and had a predominance of first-year residents. | Not stated | Not stated |  |
| Parshuram et al (2015) | Patient safety, resident well-being and continuity of carewith different resident duty schedules in the intensive care unit: a randomized trial | Canadian Medical Association Journal | To evaluate the effects of different resident duty schedules on patient safety, resident well-being, and care continuity in intensive care units (ICU) | Canada | Randomized control trial | Not specified | Not specified | Canada | Ontario | Residents from internal medicine, anesthesia, surgery and emergency medicine training programs | Mixed | Two residents participated in the schedule but did not consent to providing data for analysis | 47 | Residents from internal medicine, anesthesia, surgery and emergency medicine training programs | Not specified | Residents were randomly assigned to in-house overnight schedules of 24, 16, or 12 hours | Each resident completed a 2-month rotation | Duty hour restriction | The trial’s findings suggest that shorter duty schedules do not provide the anticipated benefits. Resident sleepiness: Assessed using the Stanford Sleepiness Scale; no significant effect of the duty schedule on sleepiness. Residents on 24-hour schedules reported more severe somatic symptoms, yet no significant differences in burnout across groups (measured using Maslach Burnout Inventory). | Potential bias due to inability to blind participants to the schedule Study may have been underpowered to detect differences in preventable adverse events Residents' adherence to schedules was not formally monitored | Canadian Institutes of Health Research, the Centre for Quality Improvement and Patient Safety of the University of Toronto, and the Research Institute at the Hospital for Sick Children | Jan Friedrich received funding to assist in completing a portion of the physician reviews of adverse events |  |
| Webb et al. 2015 | Peer mentoring for core medical trainees: uptake and impact | Postgraduate Medical Journal | To assess the uptake and impact of a peer mentoring scheme for core medical trainees on both mentors and mentees | September 2012 until August 2013 | Pre-post study | 2012-2013 | 1 year | UK | London | Core medical trainees in the Southwest London Training programme | Medicine | 23 of 42 participants completed the end-of-year questionnaire | 42 | All first or second-year core medical trainees in the Southwest London Training programme | Not specified | Trainees submitted personal statements, attended a three-session mentoring training programme on what mentorship is and how to mentor. They were matched into mentoring pairs. Each session included didactic teaching, group work, and work in pairs. Mentors and mentees decided on meeting frequency and were responsible for organizing their meetings | 1 year | Mentorship | Participating trainees viewed the scheme positively. Reported benefits included changes in their behaviour and acquiring transferable skills that might help them in later career roles, such as an educational supervisor. Reported benefits included changes in behavior, stress management, improved work relationships, and acquisition of transferable skills. 93% of trainees found the training sessions useful or very useful. No specific p values were provided in the context given. | Small size, single-specialty focus, and limited questionnaire response rate.  Lack of a standardized mentorship sessions (frequency, duration, quality of mentorship, validated approach to collecting feedback.) Some trainees did not feel 'senior enough' to take on the mentor role. | None | None |  |

Supplementary Table 1: Extraction table of studies included in the systematic review

| **Author and Year** | Selection Bias | | | | Performance Bias | | Detection Bias | | Attrition Bias | | Reporting Bias | |
| --- | --- | --- | --- | --- | --- | --- | --- | --- | --- | --- | --- | --- |
|  | Sequence generation | | Allocation concealment | | Blinding of participants and personnel. | | Blinding of outcome assessment | | Incomplete Data Outcome | | Selective Outcome Reporting | |
|  | Risk (High, Low or Unclear risk) | Justification on Judgement | Risk | Justification on Judgement | Risk | Justification on Judgement | Risk | Justification on Judgement | Risk | Justification on Judgement | Risk | Justification on Judgement |
|  |  |  |  |  |  |  |  |  |  |  |  |  |
| Brazier et al (2022) | Low | Allocation via random number generator | Unclear | Researchers were not blinded after condition assignment inorder to determine how many participants, however impact unclear | Some concerns | Participants not blinded, the effect of adhering to the interventions as specified in the trial protocol | Unclear | Not clarified whether personnel were blinded to measured primary outcome | High | If burnout or unwell, less likely to complete the survey | Low | The trial was analysed in accordance with a pre-specified plan that was finalized before unblinded outcome data were available for analysis |
| Loewenthal et al (2021) | Unclear | Not clarified how participants were allocated | High | Particpants not blinded. Control only received one introductory session. | Unclear | No blinding | Unclear | Not clarified whether personnel were blinded to measured primary outcome | Low | Drop-out justified, low number | Low | No missing data |
| Axisa et al (2019) | Low | True Random Number Generator used | High | Particpants not blinded. Control did not undergo a workshop | Unclear | No blinding | Unclear | Not clarified whether personnel were blinded to measured primary outcome | Low | Drop-out justified, low number. Higher proportion of participants were assigned to the intervention group | Low | No missing data |
| Cheung et al (2020) | Unclear | Not clarified how participants were allocated | High | Particpants not blinded to allocation group | Low | Participants blinded to primary outcome | Low | Investigators blinded to allocation. | High | 27% attrition rate | Low | No missing data |
| Arora et al (2011) | Unclear | Not clarified how participants were allocated | High | Particpants not blinded to allocation group | Unclear | Some dependent variable is objective | Unclear | Not clarified whether personnel were blinded to measured primary outcome. Some dependent variable is objective | Low | Only 10% attrition | Low | No missing data |
| Taylor et al (2020) | Unclear | Not clarified how participants were allocated | High | Particpants not blinded to allocation group | Unclear | Unclear if participants were blinded to primary outcome | Unclear | Not clarified whether personnel were blinded to measured primary outcome | Low | Only 15% attrition | Low | No missing data |
| Gunasingam et al (2015) | Unclear | Not clarified how participants were allocated | High | Particpants not blinded to allocation group | High | Particpants and personell not blinded to measured outcome | Unclear | Not clarified whether personnel were blinded to measured primary outcome | Unclear | No documentation of number of subects who dropped out | Low | Implied there is no missing data |
| Fendel et al (2021) | Low | Software Qminim used | High | Particpants not blinded to allocation group | Unclear | Unclear if participants blinded to primary outcome. | Low | Investigators blinded to allocation. | Unclear | No documentation of number of subects who dropped out | Low | Implied there is no missing data |
| Lebares et al (2019) | Unclear | Not clarified how participants were allocated | Low | Particpants blinded to allocation group | Low | Particpants blinded to allocation group | High | Personnel not blinded to allocation group or measured outcome | Unclear | No documentation of number of subects who dropped out | Low | Implied there is no missing data |
| Ireland et al (2017) | Unclear | Not clarified how participants were allocated | High | Particpants not blinded to allocation group | Unclear | Particpants and personell not blinded to measured outcome | Unclear | Personnel not blinded to allocation group or measured outcome | Unclear | No documentation of number of subects who dropped out | Low | Implied there is no missing data |
| Fraiman et al (2022) | Low | random-numbergenerator | High | Particpants not blinded to allocation group | Unclear | Unclear if participants blinded to primary outcome. | Unclear | Not clarified whether personnel were blinded to measured primary outcome | Low | 43% attrition however Characteristics between those who were lost to follow-up and those who completed the study were not markedly different | Low | Missing data justified |
| Martins et al (2011) | Unclear | Not clarified how participants were allocated | High | Particpants not blinded to allocation group, control group did not receive fake intervention | Unclear | Unclear if participants blinded to primary outcome. | Unclear | Not clarified whether personnel were blinded to measured primary outcome | Unclear | No documentation of number of subects who dropped out | Low | Implied there is no missing data |
| Lebares et al (2021) | Unclear | Not clarified how participants were allocated | High | Particpants not blinded to allocation group | Unclear | Unclear if participants blinded to primary outcome. | Unclear | Not clarified whether personnel were blinded to measured primary outcome | Low | Documented, low dropout/withdrawal rate, justified | Low | Implied there is no missing data |
| Ripp et al. (2016) | Unclear | Not clarified how participants were allocated | Low | Particpants blinded to allocation group | Unclear | Unclear if participants blinded to primary outcome. | Unclear | Not clarified whether personnel were blinded to measured primary outcome | High | 24 % incomplete data | Low | Implied there is no missing data |
| Milstein et al., (2009) | Low | Randomly assigned | High | Particpants not blinded to allocation group | Unclear | Unclear if participants blinded to primary outcome. | Unclear | Not clarified whether personnel were blinded to measured primary outcome | Low | No documentation of number of subects who dropped out - implied there were none | Low | Implied there is no missing data |
| Shea et al., 2014 | Low | Randomly assigned | High | Particpants not blinded to allocation group | Unclear | Unclear if participants blinded to primary outcome. | Low | Analysts and investigators were kept blinded | Low | Only 3/106 did not return the survey | Low | Implied there is no missing data |
| Mache et al., 2017 | Low | Randomly assigned | High | Particpants not blinded to allocation group | Unclear | Unclear if participants blinded to primary outcome. | Low | Analysts and investigators were kept blinded | Low | There were no significant differences between dropouts and those who participated at all 3 measurements | Low | balanced reporting of both successful and unsuccessful outcomes |
| Mache et al., 2016 | Low | Randomly assigned, computer generated numbers | High | Particpants not blinded to allocation group | Low | Personnel blinded | Low | The assessments were performed as self-reports through online questionnaires, and the same method of data collection was used for all participants at baseline and follow-up time points. The use of standardized instruments and consistent methods | Unclear | Participants who failed to complete the follow-up surveys did not differ in their baseline responses | Low | Implied there is no missing data |
| Bragard et al., 2009 | Low | Randomly assigned | High | Particpants not blinded to allocation group | Unclear | Unclear if participants blinded to primary outcome. | Unclear | Not clarified whether personnel were blinded to measured primary outcome | Unclear | 15% incomplete data | Low | Implied there is no missing data |
| Parshuram et al (2015) | Low | Randomly assigned | High | Particpants not blinded to allocation group | Unclear | Potential performance bias as participants mot blinded | Low | Analysts and investigators were kept blinded | Low | No dropout/withdrawal | Low | Implied there is no missing data |
| Verweij et al., 2017 | Low | Randomly assigned | Low | Particpants blinded to allocation group | Low | Adherence to CONSORT guidelines | Unclear | Not clarified whether personnel were blinded to measured primary outcome | Low | low dropout/withdrawal | Low | Implied there is no missing data |
| Saadat et al (2012) | Low | Randomly assigned | Low | Particpants blinded to allocation group | Unclear | Unclear if participants blinded to primary outcome. | Unclear | Not clarified whether personnel were blinded to measured primary outcome | Low | low dropout/withdrawal | Low | Implied there is no missing data |

Supplementary Table 2: Cochrane Risk of Bias 2 (RoB 2) tool for randomised control trials

| Study | **D1 - Risk of bias due to confounding** | | **D2 - Risk of bias arising from measurement of the exposure** | | **D3 - Risk of bias in selection of participants into the study (or into the analysis)** | | **D4 - Risk of bias due to post-exposure interventions** | | **D5 - Risk of bias due to missing data** | | **D6 - Risk of bias arising from measurement of the outcome** | | **D7 - Risk of bias in selection of the reported result** | | **Overall** |
| --- | --- | --- | --- | --- | --- | --- | --- | --- | --- | --- | --- | --- | --- | --- | --- |
|  | **Judgement** | Justification | **Judgement** | Justification | **Judgement** | Justification | **Judgement** | Justification | **Judgement** | Justification | **Judgement** | Justification | **Judgement** | Justification |  |
| Rich et al (2020) | **Some concerns** | No control | **Low** | Clear exposure | **Some concerns** | Self selecting | **Unclear** | Not clarified | **Unclear** | 18% missing data | **Low** | Validated tools used | **Low** | All reported | **Some concerns** |
| O’Riordan et al (2020) | **Some concerns** | No control | **Some concerns** | Unclear if posters have been seen by all | **Some concerns** | Self selecting | **Unclear** | Not clarified | **Very high** | 10/18 missing | **Low** | Validated tools used | **High** | Not specified that all narrative are reported | **High** |
| Bu et al (2019) | **Unclear** | ?other concurrent intervention | **Low** | Clear exposure | **Unclear** | Not stated how the 20 participants were chosen out of 28 that showed interest | **Unclear** | Not clarified | **Some concerns** | 29% returned questionnaire | **Unclear** | Non-validated tool | **Unclear** | Not able to determine | **Some concerns** |
| Stevens et al (2020) | **Low** | Cross-over | **Some concerns** | Self directed intervention | **Some concerns** | Self selecting | **Unclear** | Not clarified | **Low** | No missing data | **Low** | Validated tools used | **Low** | All reported | **Some concerns** |
| Goldhagen et al (2015) | **Unclear** | Self-selecting participants, keen to engage in therapy? | **Low** | Clear exposure | **Some concerns** | Self selecting | **Unclear** | Not clarified | **Some concerns** | 36% dropout | **Low** | Validated tools used | **Low** | All reported | **Some concerns** |
| Eisen et al (2013) | **Unclear** | Self-selecting participants, keen to engage in mentorship? | **Some concerns** | Hard to measure exposure to mentorship | **Some concerns** | Self selecting | **Unclear** | Not clarified | **Low** | No missing data | **Low** | Self reported | **Low** | All reported | **Some concerns** |
| Kashani et al (2015) | **Some concerns** | No control | **Low** | Clear exposure | **Some concerns** | Self selecting | **Unclear** | Some participants may have practiced what they have been taught post-intervention, thus affecting result | **Low** | Only 14% dropout rate | **Low** | Validated tools used | **Low** | All reported | **Some concerns** |
| Kashat et al (2020) | **Some concerns** | No control | **Low** | Clear exposure | **Low** | All eligible participants participated | **Unclear** | Some participants may have practiced what they have been taught post-intervention, thus affecting result | **Low** | No missing data | **Low** | Self reported | **Low** | All reported | **Some concerns** |
| Ospina-Kammerer et al (2003) | **Low** | Emotional exhaustion scores pre-intervention similar in both groups | **Low** | Clear exposure | **Unclear** | Not detailed how the participants were recruited | **Unclear** | Some participants may have practiced what they have been taught post-intervention, thus affecting result | **Low** | No missing data | **Low** | Self reported | **Low** | All reported | **Some concerns** |
| Zazulak et al (2017) | **Some concerns** | Control present, unclear is baseline characteristics are p>0.05 | **Low** | Clear exposure | **Some concerns** | Self selecting | **Unclear** | Some participants may have practiced what they have been taught post-intervention, thus affecting result | **Unclear** | No documentation of any missing data/dropouts | **Low** | Self reported | **Low** | All reported | **Some concerns** |
| Minichiello et al (2020) | **Some concerns** | No control | **Low** | Clear exposure | **Some concerns** | Self selecting | **Unclear** | Some participants may have practiced what they have been taught post-intervention, thus affecting result | **Unclear** | No documentation of any missing data/dropouts | **Low** | Self reported | **Low** | All reported | **Some concerns** |
| Weight et al (2013) | **Low** | P<0.05 baseline characteristics between control and intervention | **Unclear** | Self directed intervention | **Some concerns** | Self selecting participants | **Unclear** | Some participants may have practiced what they have been taught post-intervention, thus affecting result | **Unclear** | 14% dropout | **Low** | Self reported, validated instruments | **Low** | All reported | **Some concerns** |
| Romcevich et al (2018) | **Some concerns** | No control | **Low** | Clear exposure | **Some concerns** | Self selecting | **Unclear** | Some participants may have practiced what they have been taught post-intervention, thus affecting result | **Low** | No missing data | **Low** | Self reported, validated instruments | **Low** | All reported | **Some concerns** |
| Foster et al (2012) | **Some concerns** | Unclear whether participant baseline characteristics are similar | **Low** | Clear exposure | **Unclear** | Not detailed how the participants were recruited | **Unclear** | Not enough information | **Unclear** | Not enough information | **Low** | Self reported, validated instruments | **Unclear** | Not enough information | **High risk of bias** |
| Babbar et al (2019) | **Some concerns** | No control | **Unclear** | No standardisation in number of classes participants attended | **Unclear** | Not detailed how the participants were recruited | **Unclear** | Some participants may have practiced what they have been taught post-intervention, thus affecting result | **Low** | No missing data | **Low** | Self reported, validated instruments | **Low** | All reported | **Some concerns** |
| Forbes et al (2020) | **Some concerns** | P<0.05 for age, registration with GP | **Low** | Clear exposure | **Some concerns** | Self selecting | **Unclear** | Some participants may have practiced what they have been taught post-intervention, thus affecting result | **High** | High level of missing data - quantitative analysis aborted | **Low** | Self reported, validated instruments | **Unclear** | Implied qualitative results is representative | **High risk of bias** |
| Gopal et al. (2005) | **Low** | Same group of interns | **Unclear** | Possibility of recall bias | **Unclear** | Not detailed how the participants were recruited | **Low** | N/A | **Unclear** | 13-26% missing data | **Low** | Self reported, validated instruments | **Low** | All reported | **Low** |
| Barrack et al., (2006) | **Some concerns** | Different groups pre- and post work hr restriction | **Low** | Clear exposure | **Some concerns** | Self selecting participants | **Low** | N/A | **Low** | No missing data | **Low** | Self reported, validated instruments | **Low** | All reported | **Some concerns** |
| Martini et al (2006) | **Some concerns** | Different groups pre- and post work hr restriction | **Low** | Clear exposure | **Some concerns** | Self selecting participants | **Low** | N/A | **Unclear** | Only 31% response rate - significance unclear | **Low** | Self reported, validated instruments | **Low** | All reported | **Some concerns** |
| Antiel et al., 2013 | **Some concerns** | No control | **Low** | Clear exposure | **Some concerns** | Self selecting participants | **Low** | N/A | **Low** | High response rate | **Low** | Self reported, validated instruments | **Low** | All reported | **Some concerns** |
| Goitein et al., 2005 | **Unclear** | Residents were asked to isolate the effects of WHLs, but subconscious influences from other aspects of their training could affect their responses | **Low** | Clear exposure | **Some concerns** | Self selecting participants | **Low** | N/A | **Low** | High response rate | **Low** | Self reported, validated instruments | **Low** | All reported | **Some concerns** |
| Winkel et al (2010) | **Some concerns** | No control | **Low** | Clear exposure | **Some concerns** | Self selecting participants | **Unclear** | Some participants may have practiced what they have been taught post-intervention, thus affecting result | **Low** | No missing data | **Low** | Self reported, validated instruments | **Low** | All reported | **Some concerns** |
| Maher et al., 2013 | **Some concerns** | Not randomised | **Low** | Clear exposure | **Some concerns** | Self selecting participants | **Unclear** | Some participants may have practiced what they have been taught post-intervention, thus affecting result | **Low** | No missing data | **Unclear** | State Trait Anxiety Inventory and HR may not be the perfect measure of stress | **Low** | All reported | **Some concerns** |
| Runyan et al., 2016 | **Some concerns** | No control | **Low** | Clear exposure | **Some concerns** | Self selecting participants | **Unclear** | Some participants may have practiced what they have been taught post-intervention, thus affecting result | **Some concerns** | Discrepancy in the number of residents completing the pre and post measures, which could introduce bias due to attrition. | **Low** | Self reported, validated instruments | **Unclear** | The lack of statistical power means that these results should be interpreted with caution | **Some concerns** |
| Brennan et al., 2015 | **Some concerns** | Unclear whether participant baseline characteristics are similar | **Unclear** | No standardisation in number of classes participants attended | **Unclear** | no information on how they were selected | **Unclear** | Some participants may have practiced what they have been taught post-intervention, thus affecting result | **Unclear** | does not mention whether there was missing data or how missing data was handled | **Low** | used validated psychological inventories | **Unclear** | unclear if all planned analyses were reported | **Some concerns** |
| Wen et al (2017) | **Some concerns** | No control | **Unclear** | No standardised exposure | **Some concerns** | Self selecting participants | **Unclear** | Some participants may have practiced what they have been taught post-intervention, thus affecting result | **Some concerns** | 40% dropout rate | **Low** | outcomes were measured using prevalidated surveys | **Low** | no indication of selective reporting | **Some concerns** |
| Bentley et al., 2018 | **Some concerns** | No control | **Low** | Clear exposure | **Low** | All eligible participants participated | **Unclear** | Some participants may have practiced what they have been taught post-intervention, thus affecting result | **Low** | No missing data | **Low** | Self reported, validated instruments | **Low** | no indication of selective reporting | **Some concerns** |
| Taylor et al., 2016 | **Some concerns** | No control | **Low** | Clear exposure | **Some concerns** | Self selecting participants | **Unclear** | Some participants may have practiced what they have been taught post-intervention, thus affecting result | **High** | Only 11/31 completed f/u survey | **Low** | Self reported, validated instruments | **Low** | All reported | **High risk of bias** |
| Ghetti et al., 2009 | **Some concerns** | No control | **Low** | Clear exposure | **Some concerns** | Self selecting participants | **Unclear** | Some participants may have practiced what they have been taught post-intervention, thus affecting result | **Low** | No missing data | **Unclear** | Unclear whether Psychological Medicine Inventory is a validated measure | **Low** | All reported | **Some concerns** |
| McCue et al (1991) | **Some concerns** | Not randomised | **Low** | Clear exposure | **Some concerns** | Self selecting participants | **Unclear** | Some participants may have practiced what they have been taught post-intervention, thus affecting result | **Low** | No missing data | **Low** | Self reported, validated instruments | **Low** | All reported | **Some concerns** |
| Lases et al (2016) | **Unclear** | P<0.05 for academic vs non-academic in intervention and control | **Unclear** | Training of instructors not standardised | **Some concerns** | Self selecting participants | **Unclear** | Some participants may have practiced what they have been taught post-intervention, thus affecting result | **Unclear** | Higher percentage of incomplete data from control group | **Unclear** | Self reported, Likert scale used | **Low** | All available data reported | **Some concerns** |
| Chaukos et al (2018) | **Some concerns** | No control | **Low** | Clear exposure | **Some concerns** | Self selecting participants | **Unclear** | Some participants may have practiced what they have been taught post-intervention, thus affecting result | **Some concerns** | 59% dropout rate | **Low** | Self reported, validated instruments | **Low** | Negative data also reported | **High risk of bias** |

Supplementary Table 3: Risk Of Bias In Non-randomized Studies of Exposure (ROBINS-E) for observational studies of exposures

| **Study** | **Rogers et al (2016)** | **Nomura et al (2016)** | **Prins et al (2007)** |
| --- | --- | --- | --- |
| **Were the aims/objectives of the study clear?** | Yes | Yes | Yes |
| **Was the study design appropriate for the stated aim(s)?** | Unclear (Case control study) | Yes | Yes |
| **Was the sample size justified?** | Yes | Yes | Yes |
| **Was the target/reference population clearly defined? (Is it clear who the research was about?)** | No | Yes | Yes |
| **Was the sample frame taken from an appropriate population base so that it closely represented the target/reference population under investigation?** | Yes | Yes | Yes |
| **Was the selection process likely to select subjects/participants that were representative of the target/reference population under investigation?** | Yes | Yes | Yes |
| **Were measures undertaken to address and categorise non-responders?** | No | Yes - high cooperation rate, with 41 out of 42 residents | Yes |
| **Were the risk factor and outcome variables measured appropriate to the aims of the study?** | Yes | Yes | Yes |
| **Were the risk factor and outcome variables measured correctly using instruments/measurements that had been trialled, piloted or published previously?** | Yes | Yes | Yes |
| **Is it clear what was used to determined statistical significance and/or precision estimates? (e.g. p-values, confidence intervals)** | Yes | Yes | Yes |
| **Were the methods (including statistical methods) sufficiently described to enable them to be repeated?** | Yes | Yes | Yes |
| **Were the basic data adequately described?** | Yes | Yes | No |
| **Does the response rate raise concerns about non-response bias?** | No | No | No |
| **If appropriate, was information about non-responders described?** | No | No | No |
| **Were the results internally consistent?** | Unclear | Yes the quantitative and qualitative findings align with the study's objectives | Yes |
| **Were the results presented for all the analyses described in the methods?** | Yes | Yes | Yes |
| **Were the authors' discussions and conclusions justified by the results?** | Yes | Yes | Yes |
| **Were the limitations of the study discussed?** | Yes | Yes | No |
| **Were there any funding sources or conflicts of interest that may affect the authors’ interpretation of the results?** | No | Not stated | Not stated |
| **Was ethical approval or consent of participants attained?** | Yes | Yes | No |

Supplementary Table 4: Appraisal tool for Cross-Sectional Studies (AXIS)^20^ for cross sectional studies

| **Study** | **Warren et al (2021)** | **Hsu et al 2010** | **Brunworth et al (2006)** | **Feld et al (2006)** | **Webb et al. 2015** |
| --- | --- | --- | --- | --- | --- |
| **Was there a clear statement of the aims of the research?** | Yes | Yes | Yes | Yes | Yes |
| **Is a qualitative methodology appropriate?** | Yes | Yes | Yes | Yes | Yes |
| **Was the research design appropriate to address the aims of the research?** | Yes | Yes | Yes | Yes | Yes |
| **Was the recruitment strategy appropriate to the aims of the research?** | Can't tell (self-selecting) | Can't tell (self-selecting) | Can't tell (self-selecting) | Can't tell (self-selecting) | Can't tell (self-selecting) |
| **Was the data collected in a way that addressed the research issue?** | Yes | Yes | Yes | Yes | Yes |
| **Has the relationship between researcher and participants been adequately considered?** | Yes (peer conducted interview) | Unclear | Yes (anonymised web based survey) | Unclear | Unclear |
| **Have ethical issues been taken into consideration?** | Yes | Unclear | Unclear | Unclear | Yes |
| **Was the data analysis sufficiently rigorous?** | Yes | Unclear | No (not all residents exposed to same working hour restriction but data amalgamated) | Unclear | Unclear |
| **Is there a clear statement of findings?** | Yes | Unclear | Yes | Yes | Yes |
| **Is the research valuable?** | Yes | Yes | Yes | Unclear - different culture of junior doctors (20 years ago) but likely relevant | Yes |

Supplementary Table 5: Critical Skills Appraisal Programme (CASP) Qualitative Studies Checklist for qualitative studies
