# Supplementary material for: Enhancing wellbeing in medical practice: Exploring interventions and effectiveness for improving the work lives of resident (junior) doctors: A systematic review and narrative synthesis
Source: Future Healthc J. 2024 Oct 16;11(4):100195. doi: 10.1016/j.fhj.2024.100195 (PMC11584606; doi:10.1016/j.fhj.2024.100195)
Supplement: Supplementary file 2 [file mmc2.docx]

**Appendix 1**

1. "INTERVENTION*".m_titl.
2. "INITIATIVE*".m_titl.
3. NON-CONSULTANT.mp
4. "JUNIOR DOCTOR*".mp. [mp=title, book title, abstract, original title, name of substance word, subject heading word, floating sub-heading word, keyword heading word, organism supplementary concept word, protocol supplementary concept word, rare disease supplementary concept word, unique identifier, synonyms, population supplementary concept word, anatomy supplementary concept word]
5. DOCTOR*-IN-TRAINING.mp. [mp=title, book title, abstract, original title, name of substance word, subject heading word, floating sub-heading word, keyword heading word, organism supplementary concept word, protocol supplementary concept word, rare disease supplementary concept word, unique identifier, synonyms, population supplementary concept word, anatomy supplementary concept word]
6. 3 or 4 or 5
7. 1 or 2
8. 6 and 7
9. limit 8 to English language
